# Sol-gel interconvertible 62-component dodecahedra assembled via amide-π–assisted anion-coordination nanoarchitectonics

**DOI:** 10.1126/sciadv.aec5613

**Published:** 2026-06-03

**Authors:** Wen-Zheng Fu, Zhu Zhuo, Zi-Ang Nan, Wei Wang, Jun-Rui Liu, Qing Li, Lu-Yao Liu, Ting Chen, Yu-Long Zhang, Zhong-Qun Tian, Zhihao Li, Mao-Chun Hong, You-Gui Huang

**Affiliations:** ^1^ State Key Laboratory of Structure Chemistry, Fujian Institute of Research on the Structure of Matter, Chinese Academy of Science, Fuzhou, Fujian 350002, China.; ^2^ Fujian Science & Technology Innovation Laboratory for Optoelectronic Information of China, Fuzhou, Fujian 350002, China.; ^3^ University of Chinese Academy of Sciences, Beijing 100049, China.; ^4^ Xiamen Key Laboratory of Rare Earth Photoelectric Functional Materials, Xiamen Institute of Rare Earth Materials, Chinese Academy of Sciences, Xiamen, Fujian 361021, China.; ^5^ State Key Laboratory of Physical Chemistry of Solid Surfaces, MOE Key Laboratory of Spectrochemical Analysis and Instrumentation, iChEM, College of Chemistry and Chemical Engineering, College of Energy, College of Materials, College of Physical Science and Technology, Xiamen University, Xiamen, China.; ^6^ South China Advanced Institute for Soft Matter Science and Technology, School of Emergent Soft Matter, South China University of Technology, Guangzhou 510640, China.

## Abstract

Viruses achieve remarkable complexity through the self-assembly of protein subunits, yet mimicking such processes synthetically has remained a grand challenge. While a 144-component metal-organic cage has been obtained, the maximum component count for known supramolecular assemblies of weak noncovalent forces is only 20. Here, we report a 62-component supramolecular cage assembled via a bioinspired strategy. By combining anion coordination with amide-π interactions, we construct a 62-component discrete dodecahedral cage structurally analogous to the dengue virus. This synthetic architecture, comprising three distinct molecular species held together via 60 H-bonds and 30 sets of amide-π interactions, demonstrates excellent stability in solution and exhibits multiple solid structural polymorphs. A reversible sol-gel transition is achieved by a mixture of the dodecahedra in acetylacetone (acac), which is driven by the host-guest–mediated higher-order assembly. These findings establish a previously unidentified paradigm for constructing multicomponent supramolecular cages, opening avenues for developing bioinspired molecular nanocontainers and providing a foundation for diverse nanotechnological applications.

## Teaser

A 62-component dodecahedron showing reversible sol-gel transition has been assembled via amide-π–assisted anion coordination.

## INTRODUCTION

Nature’s ability to assemble intricate cage structures like virus capsids from thousands of protein subunits has long fascinated scientists (*1*–*5*). In this context, numerous supramolecular cages have been constructed via various interactions including covalent bonds (*6*, *7*), metal coordination bonds (*8*, *9*), hydrogen bonds (*10*, *11*), mechanical threading (*12*), etc. These synthetic cages show not only elegant structural artworks but also important functionalities such as catalysis (*13*), separation (*14*), and reactive species stabilization (*15*). Despite the substantial advancements achieved in this area, it remains extremely challenging to assemble large supramolecular cages comprising a lot of components. While synthetic metal-organic cages have surpassed 100 components using robust coordination bonds, replicating such complexity via noncovalent interactions, the very forces governing biological assembly, remains a grand challenge (*16*–*18*). A fundamental difficulty lies in balancing structural precision against the significant entropic penalties inherent in noncovalent systems. Consequently, prior efforts using macrocycle and nonmacrocycle molecules to construct hydrogen-bonded supramolecular polyhedra have achieved discrete cages with a maximum component count of only 20 (*19*–*24*), likely constrained by insufficient directional control and thermodynamic instability.

**Fig. 1.**
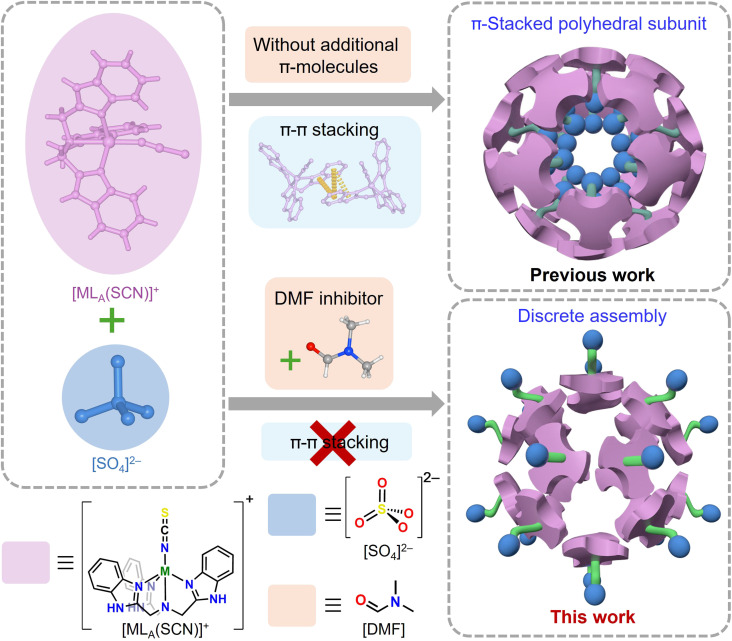
Figure showing DMF inhibiting the π-π stacking of [MLX]^+^ ions to form discrete assemblies with inverted amphiphilicity relative to the previous reported π-stacked polyhedral subunits.

Learning from the typical dodecahedral and icosahedral symmetries of intricate virus capsids (*25*), we envisaged that the engagements of tripodal [MLX]^+^ and potential pentagonal coordination SO_4_^2−^ via anion coordination (*26*–*28*) would lead to hydrogen-bonded polyhedra with complexity comparable to virus capsids. However, our long-standing attention on anion coordination–driven assembly of [MLX]^+^ (M = divalent metal ion, L = tris(2-benzimidazolylmethyl)amine, X = halogen or pseudohalide ion) achieved a series of three-dimensional hierarchical structures instead of discrete supramolecular cages (*29*–*31*). In these structures, [MLX]^+^ self-associate via π-π stacking interactions forming micelle-like π-stacked polyhedral subunits with anionophobic interior and anionophilic exterior. The π-stacked building blocks are further connected by various anions, resulting in complex hierarchical structures. Inspired by the protein folding regulated by amide-π interactions (*32*–*34*), we propose that small molecules capable of engaging in specific π-stacking interactions with the benzimidazolylmethyl arms of [MLX]^+^ could inhibit [MLX]^+^ self-association, thereby favoring the formation of discrete assemblies with inverted amphiphilicity (Fig. 1) (*35*, *36*). In this regard, dimethylformamide (DMF) is a prime candidate because of its capability of participating in amide-π interaction (*32*, *37*).

Here, we obtain a series of 62-component dodecahedral cages, the largest component count to date. They are assembled by charge-assisted H-bonds under the assistance of amide-π interactions. Isostructural cages can be assembled by different tripodal [MLSCN]^+^ scaffolds, with the same cage capable of crystallizing into different polymorphs. These cages are stable in DMF solutions, as confirmed by morphologic and spectroscopic characterizations. Furthermore, the accommodated DMF molecules can be reversibly replaced by acetylacetone (acac) molecules, leading to a reversible sol-gel transition behavior.

## RESULTS

### Synthesis and structure of Zn-H-*P*–1

The solvothermal reaction of tris(2-benzimidazolylmethyl)amine (L_A_) and Zn(SCN)_2_ in the mixture of H_2_SO_3_ aqueous solution, DMF, and CH_3_CN at 100°C for 72 hours affords a clear solution. Keeping the solution undisturbed under ambient condition for 3 days, we isolated cube-shaped crystals (**Zn-H-*P*–1**) of {[ZnL_A_SCN]_20_(SO_4_)_12_(DMF)_30_}^4−^. Single crystals of **Zn-H-*P*–1** were obtained over a broad concentration range (1 to 13 mM) for equivalent L_A_ and Zn(SCN)_2_ (fig. S1). Single-crystal x-ray diffraction (SCXRD) reveals its space group *P*–1 (table S1). The asymmetric unit of **Zn-H-*P*–1** contains a whole quasi *I*-symmetric {[ZnL_A_SCN]_20_(SO_4_)_12_(DMF)_30_}^4−^ cage (**Zn-H-cage**) (fig. S2). Herein, **Zn-H-cage** refers to an individual cage of {[ZnL_A_SCN]_20_(SO_4_)_12_(DMF)_30_}^4−^ and **Zn-H-*P*–1** refers to the crystal of **Zn-H-cage**.

**Fig. 2.**
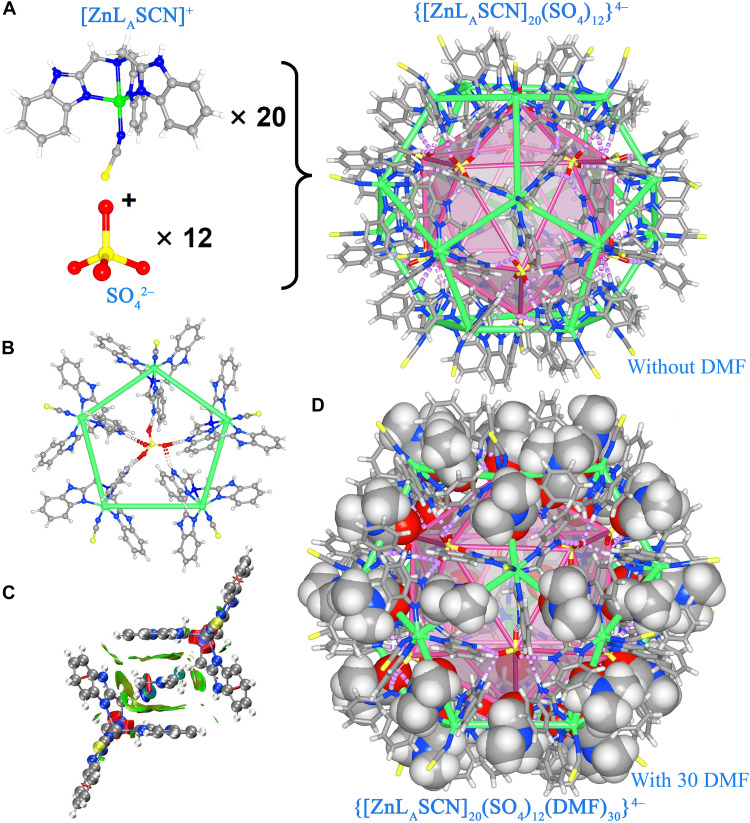
Structure of Zn-H-cage. (**A**) Formation of the dodecahedron with a small box-shaped cavity on each edge by anion coordination. (**B**) Pentagonal hydrogen-bonding mode of SO_4_^2−^. a.u., arbitrary units. (**C**) Sandwich-type amide-π interaction within the box cavity of **Zn-H-cage**. (**D**) A 62-component cage accommodating 30 DMF molecules with a hydrophilic interior and a hydrophobic shell.

For **Zn-H-cage**, 20 [ZnL_A_(SCN)]^+^ ions occupying the 20 vertexes are connected by 12 SO_4_^2−^ forming an approximately spherical dodecahedron with a small box-shaped cavity on each edge (Fig. 2A). Each SO_4_^2−^ locates in each facet showing a pentagonal hydrogen-bonding mode (Fig. 2B). Each box cavity accommodates one DMF molecule via a set of sandwich-type amide-π interactions leading to a 62-component cage (Fig. 2D), which mirrors the structure of dengue virus (fig. S3) (*25*) and C_20_-fullerene (fig. S4). As revealed by density functional theory (DFT) calculations, CH-O, CH-π, and amide-π interactions can be detected within the sandwich motif, giving an energy of −6.49 kcal/mol between each DMF and each benzimidazoylmethyl arm (Fig. 2C). The **Zn-H-cage** represents the highest component count. It is also the first fullerene-type supramolecular cage assembled via anion coordination. The 30 sets of amide-π interactions within **Zn-H-cage** are critical for the stabilization of crystalline **Zn-H-*P*–1**. As revealed by powder x-ray diffraction (PXRD) (fig. S5), the crystal structure of **Zn-H-*P*–1** collapsed upon desolvation.

All [ZnL_A_SCN]^+^ scaffolds in the dodecahedral cage show the same handedness, making each single cage homochiral (fig. S6). It is worth noticing that DMF molecules inhibit the self-association of [ZnL_A_(SCN)]^+^ via π···π interactions. This is critical to the formation of this dodecahedron. Within the dodecahedron, the amine group of all [ZnL_A_(SCN)]^+^ and the amide group of all DMF molecules are oriented toward the center of the polyhedron, and the icosahedron defined by 12 SO_4_^2−^ anions is embedded into the dodecahedron defined by [ZnL_A_(SCN)]^+^ and DMF, resulting in a hydrophilic interior and a hydrophobic shell. The 3.3-nm-diameter circumscribed and 1.5-nm-diameter inscribed spheres define the outer and inner shells of the dodecahedral cage (fig. S7). The hydrophilic interior, with a volume of ~1700 Å^3^, is filled with disordered solvent molecules, likely with H_3_O^+^ as the counter ions. In the way of equal-diameter sphere packing, **Zn-H-cages** show AAA··· quasi-simple-hexagonal-packing in the crystal structure of **Zn-H-*P*–1** (fig. S8). Both enantiomers of **Zn-H-cages** exist in the lattice leading to a racemic crystal structure.

### Synthesizing isostructural cages with diverse [MLSCN]^+^ and exploiting polymorphs of the same cage

By varying the metal ion in the synthesis, isostructural supramolecular cages can be obtained. The solvothermal reaction of L_A_ and Co(SCN)_2_ under similar conditions yields an isostructural {[CoL_A_SCN]_20_(SO_4_)_12_(DMF)_30_}^4−^ cage (**Co-H-cage**), which crystallizes in three different phases: **Co-H-*P–*1**, **Co-H-*R–*3*c***, and **Co-H-*Pba*2** (tables S2 to S4 and figs. S9 to S11). Furthermore, isostructural supramolecular cages can be obtained by using different tritopic amine ligands. The solvothermal reaction of methylbenzimidazolylmethyl)amine (L_B_) and Zn(SCN)_2_ under similar condition leads to the formation of isostructural {[ZnL_B_SCN]_20_(SO_4_)_12_(DMF)_30_}^4−^ cage (**Zn-CH**_**3**_**-cage**), crystallizing in the **Zn-CH**_**3**_**-*Pcca*** and **Zn-CH**_**3**_**-*C*2*/c*** phases (tables S5 and S6 and figs. S12 and S13). Similarly, the solvothermal reaction of tris(2-dimethylbenzimidazolylmethyl)amine (L_C_) and Zn(SCN)_2_ affords an isostructural [ZnL_C_SCN]_20_(SO_4_)_12_(DMF)_30_}^4−^ cage (**Zn-(CH**_**3**_**)**_**2**_**-cage**), which crystallizes in the phase of **Zn-(CH**_**3**_**)**_**2**_**-*Fd–*3*c*** and **Zn-(CH**_**3**_**)**_**2**_**-*Pba*2** (tables S7 and S8 and figs. S14 and S15). In particular, the lattice parameter for **Zn-(CH**_**3**_**)**_**2**_**-*Fd–*3*c*** is *a* = 93.1346 Å, which is close to single crystals of small protein molecules (*38*). Both enantiomers of the cages exist in the lattices of all eight phases (figs. S6, S16, and S17), making them racemic. The packing patterns of the cages in lattices are shown in fig. S8.

### Solution stability of Zn-H-cage

**Fig. 3.**
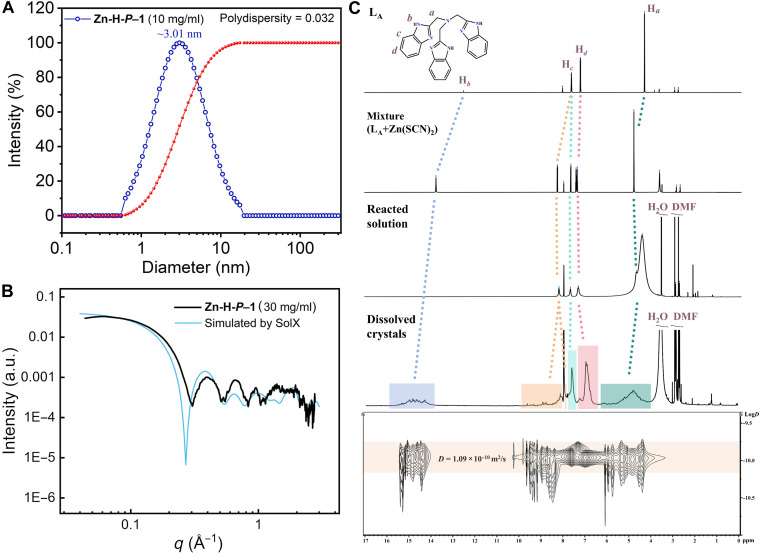
Characterizations of Zn-H-cage in DMF. (**A**) Size distribution of **Zn-H-*P–*1** in DMF at 10 mg/ml analyzed by DLS. (**B**) Simulated and experimental SAXS patterns of **Zn-H-*P–*1** (30 mg/ml) in DMF. (**C**) ^1^H NMR spectra for the solution of ligand L_A_, the solution of the mixture of L_A_ and Zn(SCN)_2_, the reacted solution after thermal treatment for the synthesis of **Zn-H-*P*–1**, and the solution of **Zn-H-*P–*1** (the solvents for all the solutions are *d*_7_-DMF).

To verify the solution stability of the dodecahedra, we initially characterized **Zn-H-cage** in DMF by dynamic light scattering (DLS) and small-angle x-ray scattering (SAXS). DLS measurements revealed the presence of nanoparticles with an average diameter of ~3.01 nm (Fig. 3A), which is close to the size of **Zn-H-cage** observed by SCXRD. The SAXS profiles of the **Zn-H-cage** solutions over a series of concentrations also match well with the simulated scattering patterns by Guinier analysis (*39*), confirming the existence of spherical particles of 2.98 nm in diameter. The SAXS result is also consistent with the **Zn-H-cage** size observed by SCXRD, confirming the stability of **Zn-H-cage** in DMF (Fig. 3B and figs. S18 to S20). Furthermore, the results obtained by pair distance distribution function analysis (*40*) are consistent with those by Guinier analysis (fig. S21 and table S10). On the other hand, **Zn-H-cage** disassociates in dimethyl sulfoxide (DMSO) as revealed by SAXS measurements (fig. S22). In this solvent, the species recrystallized into a porous structure {[ZnL_A_SCN]_4_(SO_4_)_2_·(DMF)_4_·5H_2_O}_n_ (**1**) in which anion coordination, π-π interaction, and amide-π interaction exist (table S9 and figs. S23 and S24). In the structure of **1**, each DMF interacts with one benzimidazoylmethyl arm. As a result, [ZnL_A_SCN]^+^ self-associate via π-π interactions to form zigzag chains (fig. S24D) rather than being connected to a closed polyhedron by SO_4_^2−^ and DMF.

To further verify the solution status of the supramolecular cage, we performed ^1^H nuclear magnetic resonance (NMR) measurements on four solutions: a solution of ligand L_A_ in *d*_7_-DMF (fig. S25), a mixture of L_A_ and Zn(SCN)_2_ in *d*_7_-DMF (fig. S26), the reacted solution after thermal treatment for synthesizing **Zn-H-*P–*1** (fig. S27), and a dissolved sample of crystalline **Zn-H-*P–*1** in *d*_7_-DMF (figs. S28 to S31). Compared with the highly symmetric proton signals of ligand L_A_, a set of split resonances were observed for both the reacted solution for synthesizing **Zn-H-*P–*1** and dissolved **Zn-H-*P–*1** in *d*_7_-DMF (Fig. 3C), suggesting a remarkable level of structural complexity. The broadness and desymmetrization of H_a_ and H_b_ for these two solutions may be ascribed to their complicated H-bonds with SO_4_^2−^ and solvent molecules. Unexpectedly, significant differences between the spectra of the reacted solution and the solution of dissolved crystals were observed. These differences may be ascribed to the fact that only well-defined cages exist in the latter solution while various fragments of the cage exist in the former. On the other hand, active H exchange may occur between H_2_O (introduced by the reactant H_2_SO_3_ aqueous solution) and the species with L_A_ in the reacted solution, which may also lead to a significant difference (fig. S32). Diffusion-ordered ^1^H NMR [diffusion-ordered spectroscopy (DOSY)] revealed a diffusion band at the diffusion coefficient *D* = 1.32 × 10^−10^ m^2^ s^−1^ for the reacted solution (fig. S33) and *D* = 1.09 × 10^−10^ m^2^ s^−1^ for the dissolved **Zn-H-*P–*1** (Fig. 3C and fig. S34), corresponding to average particle diameters of ~2.2 and ~3.0 nm, respectively. Meanwhile, we did not observe DMF molecules diffusing with **Zn-H-cage**, which can be attributed to the possible rapid exchange between cage-bound DMF and the bulk *d*_7_-DMF solvent. The NMR evaluated species size for the solution of dissolved crystals is consistent with that of **Zn-H-cage** observed by SCXRD, but that for the reacted solution is obviously smaller than that of **Zn-H-cage** also implying that various fragments of the cage exist in the solution.

Last, we analyzed the reacted solution and DMF solution of dissolved crystals using high-resolution electrospray ionization mass spectrometry (HR-ESI-MS) measurement. A signal corresponding to the cage without DMF molecule {[ZnL_A_SCN]^+^_20_[(SO_4_)^2−^]_12_ + [(SO_4_)^2−^]_2_ + 5H^+^+12H_2_O}^3−^ was detected for the solution of dissolved crystals (fig. S35), indicating that H-bonds are stronger than amide-π interactions allowing DMF molecules escaping from the box-shaped cavities on the edges. On the other hand, species of {[ZnL_A_SCN]^+^_20_[(SO_4_)^2−^]_12_ + [(SO_4_)^2−^]_3_ + 6H^+^+6H_2_O + DMF}^4−^ and {[ZnL_A_SCN]^+^_20_[(SO_4_)^2−^]_12_ + [(SO_4_)^2−^]_4_ + 8H^+^+8H_2_O}^4−^ were detected for the reacted solution (fig. S36), which indicates that well-defined cages exist in the solution as well besides various fragments of the cage.

### Sol-gel transition of Zn-H-cage in acac

**Fig. 4.**
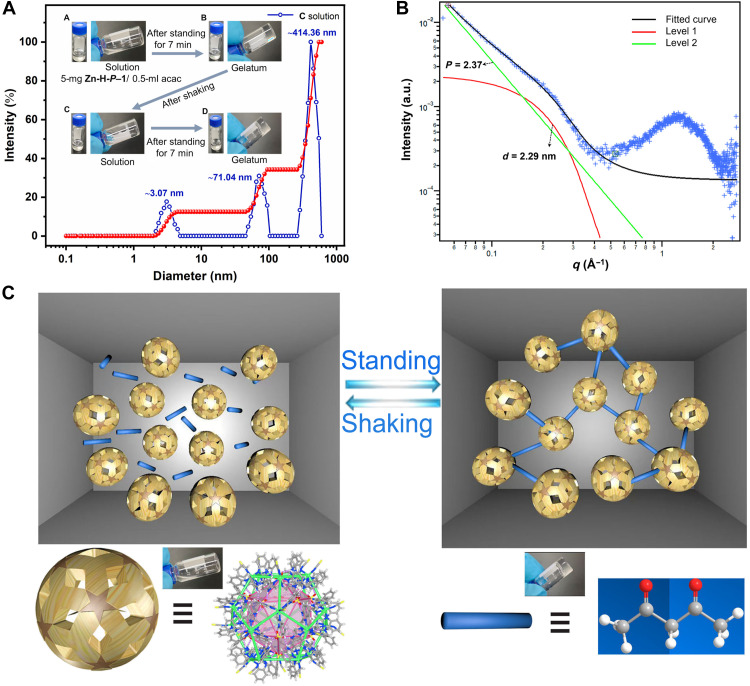
Characterizations of the sol-gel transition of Zn-H-cage in acac. (**A**) Size distribution of the solution of **Zn-H-cage** in acac analyzed by DLS (inset: photographs showing the reversible sol-gel transition process). (**B**) Simulated and experimental SAXS patterns of the gel of **Zn-H-cage** in acac. (**C**) Proposed mechanism of the sol-gel transition of **Zn-H-cage** in acac.

Since the encapsulated DMF molecules can be readily released from the polyhedra, we aimed to exploit dynamic host-guest interactions to obtain stimulus-responsive materials. The solubility of **Zn-H-P–1** was assessed by sonicating 5 mg of crystals in 0.5 ml each of 29 different solvents (fig. S37). **Zn-H-*P*–1** shows great solubility in several solvents such as pyridine, *N*-methylpyrrolidone, etc. (fig. S37B). To assess the stability of **Zn-H-cage** in these solvents, we collected the resulting solutions of **Zn-H-*P*–1** in some of these solvents such as D_2_O, acetone-*d*_6_, etc. by filtration and used them for ^1^H NMR measurements (fig. S38). The results showed that the solutions of **Zn-H-*P*–1** in acetone-*d*_6_ and acac show similar spectra to that of the solution of **Zn-H-*P*–1** in DMF, implying that **Zn-H-cage** probably remains intact in these solvents and can accommodate these solvent molecules. Unfortunately, we failed to obtain single crystals from these solvents. A reversible sol-gel transition was observed when **Zn-H-*P*–1** was dissolved in acac (fig. S37C). Initially, dissolution yielded a clear solution, which transformed into a gel after standing undisturbed for 7 min. The resulting gel reverted to a solution upon vigorous shaking, and this transition could be repeated over multiple cycles (Fig. 4A, inset).

Signals corresponding to the intact cage with one acac was detected via HR-ESI-MS (fig. S39), confirming that **Zn-H-cages** did not disassociate in acac. This was also supported by DLS measurement, which identified nanoparticles with a diameter of ~3.07 nm (Fig. 4A). Furthermore, diffusion-ordered ^1^H NMR (DOSY) of **Zn-H-*P*–1** in acac revealed a diffusion band at the diffusion coefficient *D* = 1.24 × 10^−10^ m^2^/s corresponding to a particle diameter of ~3.04 nm (fig. S40). The SAXS profile of the gel was well fitted using the Guinier-Porod model (*39*–*44*), likely indicating a mass fractal composed of spherical particles with a diameter of ~2.29 nm (Fig. 4B). In addition, transmission electron microscopy characterization revealed a morphology composed of spherical particles that is consistent with the proposed microstructure (fig. S41).

On the basis of these observations, we proposal that the reversible sol-gel transition is driven by dynamic host-guest interactions (*45*) between **Zn-H-cage** and guest molecules. Because of the double carbonyl groups, acac acts as a ditopic linker capable of connecting **Zn-H-cages** into supramolecular polymers (Fig. 4C). A model of one acac molecule bridging two individual cages is proposed in fig. S42. As revealed by DFT calculation, each acac occupies two cavities from two individual cages, giving an energy of −1.43 kcal/mol between each carbonyl group and each benzimidazoylmethyl arm. Although the π-π stacking interaction between acac and the benzimidazoylmethyl arms of ligand L_A_ is much weaker than the amide-π interaction present in the parent **Zn-H-cage**, the encapsulated DMF molecules still can be readily displaced by acac molecules in acac solution considering the exchange balance. On the other hand, the captured acac may escape from the cavities triggered by external stimuli due to the labile binding. This dynamic binding between **Zn-H-cage** and acac allows the linkage to be readily disrupted under mechanical shaking and reformed upon standing, facilitating the reversible sol-gel transition.

**Fig. 5.**
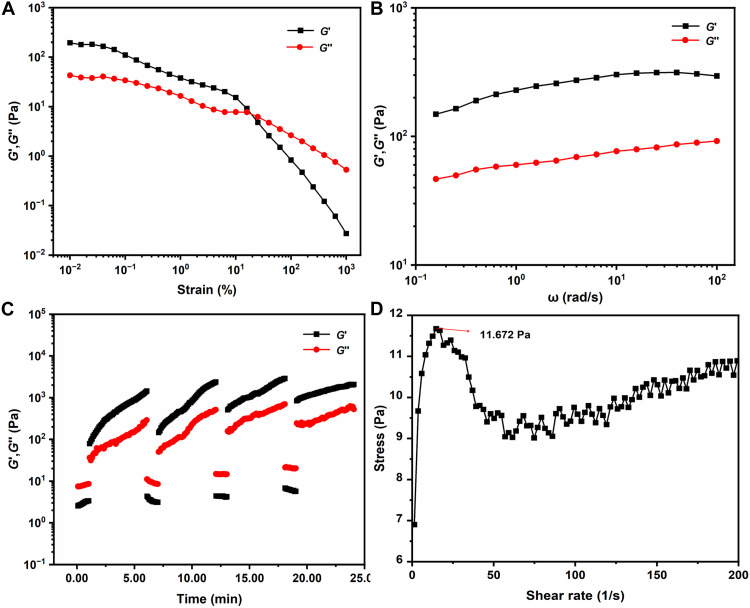
Rheological experiments of the gel of Zn-H-cage in acac. (**A**) Storage modulus (*G*′) and loss modulus (*G*″) of the gel versus strain sweep (%). (**B**) Storage modulus (*G*′) and loss modulus (*G*″) versus frequency (ω) for the gel. (**C**) *G*′ and *G*″ of the gel in continuous step-strain measurements that 100% strain for 60 s was applied, and then the strain was released to 0.05% for 300 s; 4 cycles were completed. (**D**) Stress of the gel versus shear rate.

To give insights into the properties of the resulting gel, we performed rheological experiments to characterize the gel. Strain sweep results indicated that an elastomer response (the storage modulus *G*′ > the loss modulus *G*″) was reflected before the critical strain zone (γ = 16.4%); after that, *G*′ < *G*″ was observed, which can be attributed to the disassembly of the gel (Fig. 5A). Frequency sweep measurements revealed that *G*′ was larger than *G*″ over a wide range of frequencies (Fig. 5B), which represented the quasi-solid property of the gel (*46*). The viscosity of the gel decreased with the frequency increase (fig. S43). Subsequently, the gel was subjected to large (100%) and little (0.05%) strains, respectively. When the gel was kept for under 100% strain for 60 s, *G*′ was below *G*″, suggesting that the gel was broken. Then, both *G*′ and *G*″ almost restored to their original values when the strain was reverted to 0.1% (Fig. 5C). There was no obvious loss of modulus among the repeated process for 4 cycles, implying excellent self-healing properties of the gel. The critical stress of the gel is 11.672 Pa as indicated by the stress-shear rate curve (Fig. 5D). The gel kept intact after sonication for 30 min (fig. S44) and can be disassembled by heating at 70°C for 10 min (fig. S45). In addition, the gel was disassembled after being squeezed by plastic injector and recovered after standing undisturbed (fig. S46).

## DISCUSSION

This study presents a bottom-up exploration of a series of dodecahedra, the highest component-count and first fullerene-type structures assembled via anion coordination. Each box-shaped cavity, located on the edges of these dodecahedra, accommodates a DMF molecule via sandwich-type amide-π interactions, resulting in 62-component virus-like assemblies. In addition to crystallizing in multiple phases, the dodecahedral cages remain stable and discrete in DMF solution. Notably, a reversible sol-gel transition was achieved by mixing **Zn-H-cage** with acac, facilitated by dynamic carbonyl-π interactions between the cage and acac. These findings mark a milestone in exploring anion-coordinated supramolecular cages to imitate biological assemblies, spanning controlled amphiphilicity, stimulus-responsive behavior, and potential molecular delivery functions. In specific, smart molecular delivery nanoarchitectonics may be envisaged by integrating both molecular encapsulation ability and stimulus-responsive property into an individual supramolecular cage described in this work.

## MATERIALS AND METHODS

### Starting materials

Nitrilotriacetic acid (C_6_H_9_NO_6_, 99%, Adamas), *O*-phenylenediamine (C_6_H_8_N_2_, 99%, Adamas), 3,4-diaminotoluene (C_7_H_10_N_2_, 98%, Adamas), and 4,6-dimethyl-1,2-phenylenediamine (C_8_H_12_N_2_, >98%, Adamas) were used for the syntheses of ligands L_A_, L_B_, and L_C_ according to the procedure reported in literatures (*47*–*49*). Potassium thiocyanate (KSCN, >98.5%, Greagent) and zinc nitrate hexahydrate [Zn(NO_3_)_2_·6H_2_O, >99%, Greagent] were used for the synthesis of zinc thiocyanate [Zn(SCN)_2_] according to the procedure reported in literature (*50*). Anhydrous Co(SCN)_2_, H_2_SO_3_ aqueous solution, and all the solvents were commercially obtained and directly used without further purification.

### Syntheses of compounds

#### *Synthesis of Zn-H-*P*–1*

Typically, ligand L_A_ (0.050 g, 0.123 mmol), Zn(SCN)_2_ (0.037 g, 0.206 mmol), 2 ml of DMF, 8 ml of acetonitrile, and 1.2 ml of H_2_SO_3_ aqueous solution (12.6 M) were added in a 20-ml glass vial. After ultrasonicating for 10 min, the mixture was heated at 100°C for 72 hours and then cooled to 30°C in 48 hours. The resulting mixture was kept undisturbed under the ambient condition for 72 hours, and colorless crystals of **Zn-H-*P*–1** were obtained. Yield: 55% based on L_A_ ligand. Single crystals of **Zn-H-*P*–1** can be obtained over a broad concentration range (1 to 13 mM) for both L_A_ and Zn(SCN)_2_.

#### *Synthesis of the mixed phases of Co-H-*P*–1, Co-H-*R*–3*c*, and Co-H-*Pba*2*

Ligand L_A_ (0.050 g, 0.123 mmol), Co(SCN)_2_ (0.050 g, 0.285 mmol), 2 ml of DMF, 8 ml of acetonitrile, and 1.2 ml of H_2_SO_3_ aqueous (12.6 M) were added in a 20-ml glass vial. After ultrasonicating for 10 min, the mixture was heated at 100°C for 72 hours and then cooled to 30°C in 48 hours. The resulting mixture was kept undisturbed under the ambient condition for 72 hours, and purple crystals of the mixture of **Co-H-*P*–1**, **Co-H-*R–*3*c***, and **Co-H-*Pba*2** were obtained. Yield: 50% based on L_A_ ligand.

#### *Synthesis of the mixed phases of Zn-CH_3_-*P*cca and Zn-CH_3_-*C*2/*c

Ligand L_B_ (0.060 g, 0.134 mmol), Zn(SCN)_2_ (0.037 g, 0.206 mmol), 2 ml of DMF, 8 ml of acetonitrile, and 1.2 ml of H_2_SO_3_ aqueous (12.6 M) were added in a 20-ml glass vial. After ultrasonicating for 10 min, the mixture was heated at 100°C for 72 hours and then cooled to 30°C in 48 hours. The resulting mixture was kept undisturbed under the ambient condition for 72 hours, and colorless crystals of the mixture of **Zn-CH**_**3**_**-*P*cca** and **Zn-CH**_**3**_**-*C*2/*c*** were obtained. Yield: 66% based on L_B_ ligand.

#### *Synthesis of the mixed phases of Zn-(CH_3_)_2_-*Fd*–3*c *and Zn-(CH_3_)_2_-*Pba*2*

Ligand L_C_ (0.060 g, 0.123 mmol), Zn(SCN)_2_ (0.037 g, 0.206 mmol), 2 ml of DMF, 8 ml of acetonitrile, and 1.2 ml of H_2_SO_3_ aqueous (12.6 M) were added in a 20-ml glass vial. After ultrasonicating for 10 min, the mixture was heated at 100°C for 72 hours and then cooled to 30°C in 48 hours. The resulting mixture was kept undisturbed under the ambient condition for 72 hours, and colorless crystals of the mixture of **Zn-(CH**_**3**_**)**_**2**_**-*Fd*–3*c*** and **Zn-(CH**_**3**_**)**_**2**_**-*Pba*2** were obtained. Yield: 54% based on L_C_ ligand.

#### Synthesis of {[ZnL_A_SCN]_4_(SO_4_)_2_·(DMF)_4_·5H_2_O}_n_ (1)

**Zn-H-*P*–1** (30 mg) and 1 ml of DMSO were added in a 5-ml glass vial. After ultrasonicating for 10 min, a colorless and clear solution was obtained. The resulting solution was kept undisturbed for 96 hours, and colorless block-shaped crystals of **1** were obtained.

## Supplementary Materials

### This PDF file includes:

Supplementary Text Figs. S1 to S46 Tables S1 to S10 References
