# Supplementary material for: Sol-gel interconvertible 62-component dodecahedra assembled via amide-π–assisted anion-coordination nanoarchitectonics
Source: Sci Adv. 2026 Jun 3;12(23):eaec5613. doi: 10.1126/sciadv.aec5613 (PMC13232590; doi:10.1126/sciadv.aec5613)
Supplement: Supplementary file 1 — Supplementary Text Figs. S1 to S46 Tables S1 to S10 References [file sciadv.aec5613_sm.pdf]

Science Advances
AAAS

Supplementary Materials for

Sol-gel interconvertible 62-component dodecahedra assembled via amide-π–assisted anion-coordination nanoarchitectonics

Wen-Zheng Fu *et al.*

Corresponding author: Zhu Zhuo, zhuozhu@fjirsm.ac.cn; Zi-Ang Nan, nanziang@fjirsm.ac.cn; You-Gui Huang, yghuang@fjirsm.ac.cn

Sci. Adv. **12**, eaec5613 (2026)
DOI: 10.1126/sciadv.aec5613

This PDF file includes:

Supplementary Text
Figs. S1 to S46
Tables S1 to S10
References

Supplementary Text

Crystallography. Single-crystal X-ray data for all the compounds were harvested on a XtaLAB Synergy R, HyPix diffractometer with Cu-*Kα* radiation at 100 K. The structures were solved by ShelXT with intrinsic phasing and refined on F^2 using full-matrix least-squares methods, with ShelXL and Olex (*51*) used as graphical user interfaces. Due to the huge voids of the structures, some geometrical constraints were applied (see the CIF files). The routine SQUEEZE was used to remove the very diffuse electron density associated with some of the highly disordered solvent molecules. The X-ray crystallographic coordinates for the structures reported in this article has been deposited at the Cambridge Crystallographic Data Centre (CCDC), under deposition numbers CCDC 2456179, 2456055, 2456059, 2456058, 2456066, 2456065, 2456062, 2456063, and 2456060. This data can be obtained free of charge from The Cambridge Crystallographic Data Centre via www.ccdc.cam.ac.uk/data_request/cif. Crystallographic details are listed in Tables S1–S9.

Small angle X-ray scattering (SAXS). SAXS data were collected on an Xeuss 3.0 SAXS/WAXS instrument with an 8.05 keV Cu-*Kα* radiation source (λ = 1.54189 Å). The instrument is equipped with an Eiger2R 1M detector, containing pixels of 75×75 μm^2 in size. The sample-to-detector distance is 80 mm. DMF solutions containing **Zn-H-*P*–1** at different concentrations, were measured in glass capillaries. The blank solvent (i.e. DMF) was also measured as the background sample. To increase the signal to noise ratio, the data collection process was conducted for 300 seconds. All measurements were done at 24 °C with the vacuum level of the topical path maintained below 1 mbar during the measurement. The Xenocs XSACT software was used for data collection, treatment, and preliminary analysis (i.e. primary beam removal, integration, and background subtraction). A simulated SAXS pattern for the **Zn-H-cage** was generated from its .xyz structure file using the SolX software. The radius of gyration (*Rg*) was derived via Guinier analysis, and the cage diameter was determined by fitting the pattern to a sphere model; both procedures were conducted using the IRENA package (*41*) within IgorPro 9. From the corresponding experimental SAXS data, the pair distance distribution function (PDDF) was extracted using the Moore method (*40*) in IRENA over a q-range of ~0.03–0.85 Å^{-1}. For direct structural comparison, the simulated PDDF profile was then generated by applying the same PDDF analysis to the SolX-generated pattern of the **Zn-H-cage**. Rg values obtained from different analysis methods were listed in Table S10.

Dynamic light scattering (DLS). DLS analysis was performed on Particle Size and Zeta Potential Analyzer (Brookhaven NanoBrook Omni).

Nuclear magnetic resonance (NMR). ^{1}H NMR measurements (Dosy) were performed on JEOL JNM-ECZ600R at 298 K, and the other NMR measurements were performed on Quantum-I Plus 600 MHz. DOSY ^{1}H NMR spectra were processed in Mestrenova, and solvodynamic radii (r_s) was calculated using the Stokes–Einstein equation $D = k_B T / 6\pi\eta f r_s$. T is sample temperature, k_B is Boltzman constant. The viscosity of the solutions (η) was estimated using the viscosity of non-deuterated DMF (0.92×10^{-3} kg m^{-1} s^{-1}) (*52*). For large molecules such as **Zn-H-cage**, f (factor) equals to 1. The values of D (reacted solution), D (dissolved crystals in d_7-DMF) and D (dissolved crystals in acac) are estimated to be $1.32 \times 10^{-10} \pm 1.26 \times 10^{-12}$ m^2/s, $1.09 \times 10^{-10} \pm 7.67 \times 10^{-12}$ m^2/s, and $1.24 \times 10^{-10} \pm 4.57 \times 10^{-12}$ m^2/s respectively, corresponding to radii of 1.79 ± 0.02 nm, 2.18 ± 0.15 nm, and 1.91 ± 0.07 nm. Then we further used the measured D value for DMF as an internal reference to estimate solvodynamic radii of the species in the three different solutions, and f factor was corrected by $f_{\mathrm{GW}} = \left(\frac{3r_s}{r} + \frac{r}{r+r_s}\right)^{-1}$ (1) proposed by Gierer

and Wirtz (*53*) (r and r_s represent hydrodynamic radiis for the solute and solvent, respectively). The molecular weight of solute i (MW_i) was related to its hydrodynamic radius according to $r_i = \sqrt[3]{\frac{3*MW_i}{4\pi N_A \rho}}$ (2), where N_A is Avogadro's number and ρ is the solute density. $r_s/r = \sqrt[3]{MW_S/MW}$ (r and r_s represent the hydrodynamic radiis for DMF and d_7-DMF) and $D_{DMF} \times f_{GW} \times r_{DMF} = D_{cage} \times r_{cage}$ can be concluded over here, and the corrected diameters for the species in the reacted solution, the solution of dissolved crystals in d_7-DMF, and the solution of dissolved crystals in acac were estimated to be 2.24 ± 0.02 nm, 3.02 ± 0.2 nm, and 3.04 ± 0.12 nm, respectively.

High-resolution mass spectrometry (HRMS). High Resolution Electrospray Ionization Mass Spectrometry (HR-ESI-MS) was performed on an Agilent Technologies ESI-TOF-MS.

Powder X-ray diffraction (PXRD). PXRD measurement was performed on a Rigaku SmartLab diffractometer with Cu-K_α radiation.

Transmission Electron Microscope (TEM). TEM images were obtained using JEM-F200.

Rheological Experiments. Rheological experiments of the gel were performed on TA Instrument Dynamic Hybrid Rheology 2 (DHR – 2) at 298 K. The concentration of the measured gel was 10 mg/mL, and the diameter of the measured sample was 25 mm.

DFT calculations. The Interaction Region Indicator (IRI) calculations for the amide–π interaction and π–π interaction were performed using the Gaussian 16 program (*54*) with the M06 function. In the calculations, the 6-31G* basis set was used for C, H, N, and S atoms, while the pseudopotential LANL2DZ basis set was applied to the Co atom. The IRI analyses have been performed by the Multiwfn package (*55*). BSSE-corrected interaction energies between fragment $[CoLSCN]^+$ and DMF and acac were obtained by B3LYP/6-31G* calculations.

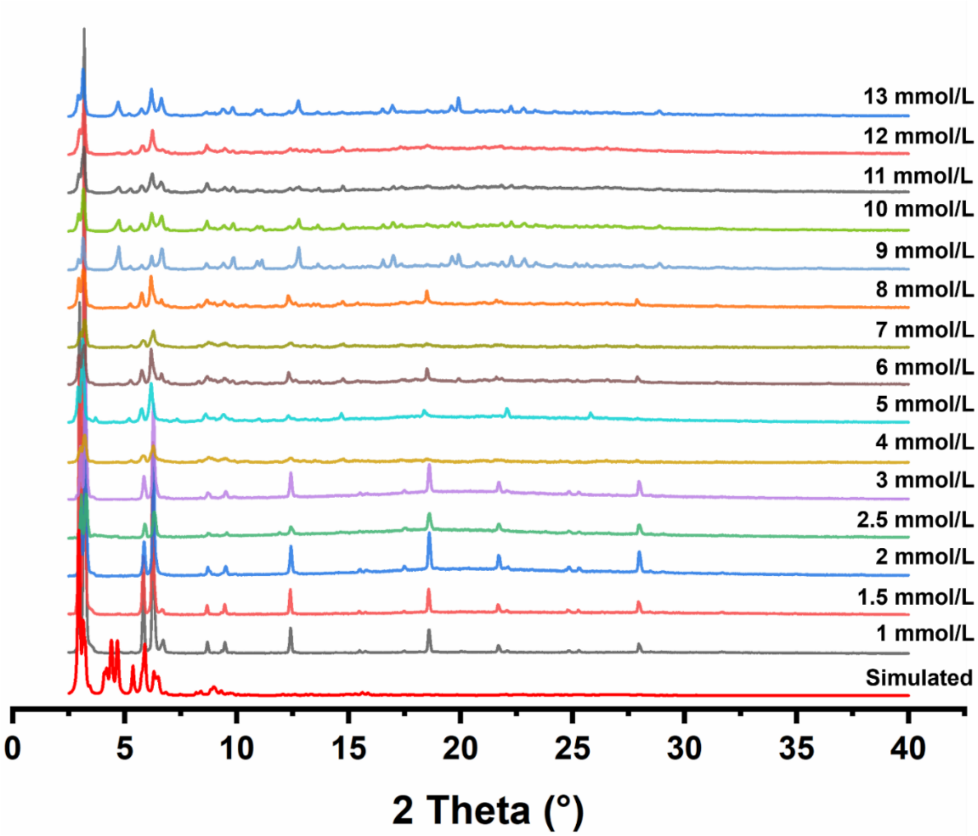

Fig. S1. PXRD patterns for the crystals obtained from the syntheses with L_A and $Zn(SCN)_2$ at different concentrations.

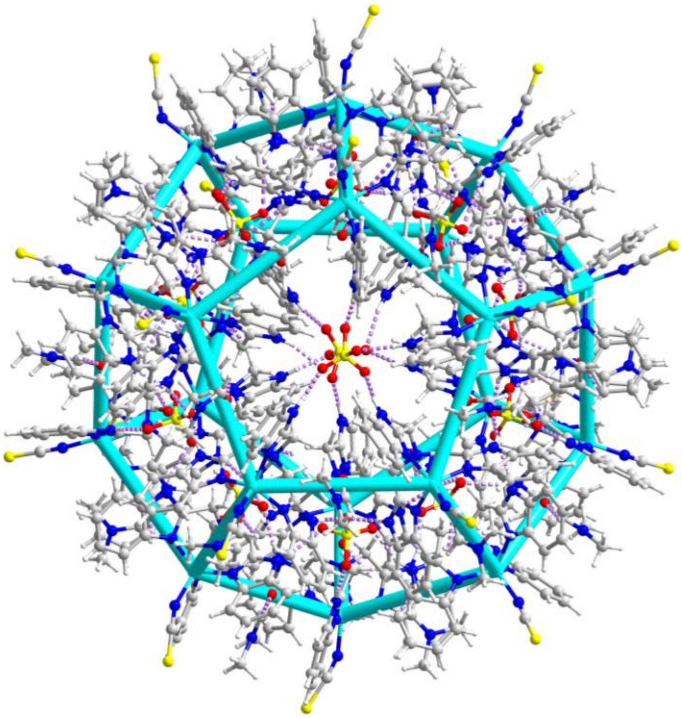

Fig. S2. Asymmetry unit of **Zn-H-*P*–1** containing an intact **Zn-H-cage**.

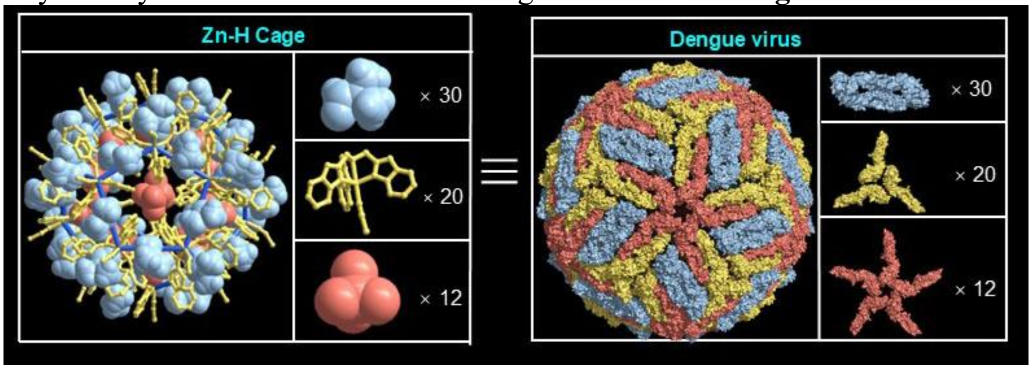

Fig. S3. Comparison of the structure of **Zn-H-cage** with dengue virus.

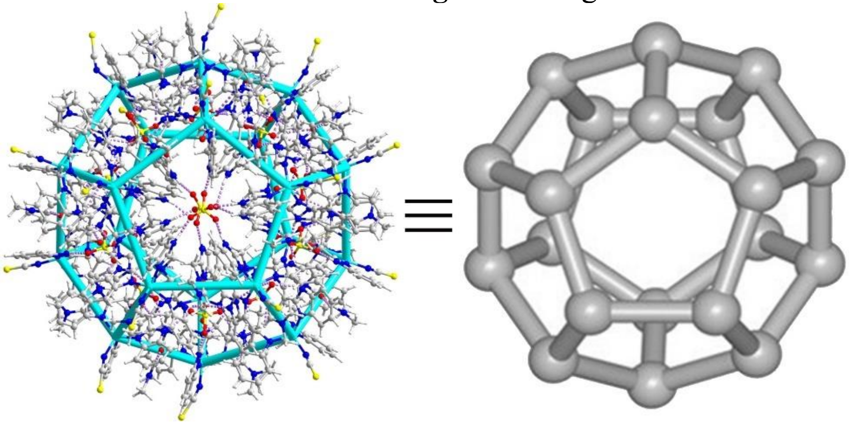

Fig. S4. Geometry comparison of **Zn-H-cage** with C_{20}-fullerene.

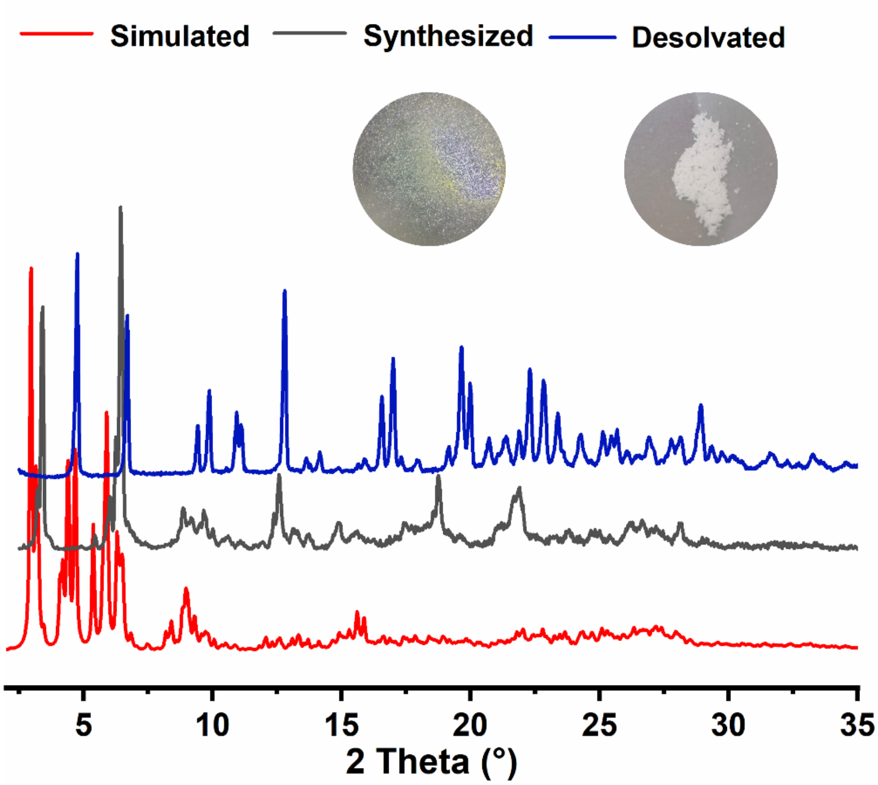

Fig. S5. PXRD patterns of **Zn-H-*P*–1**.

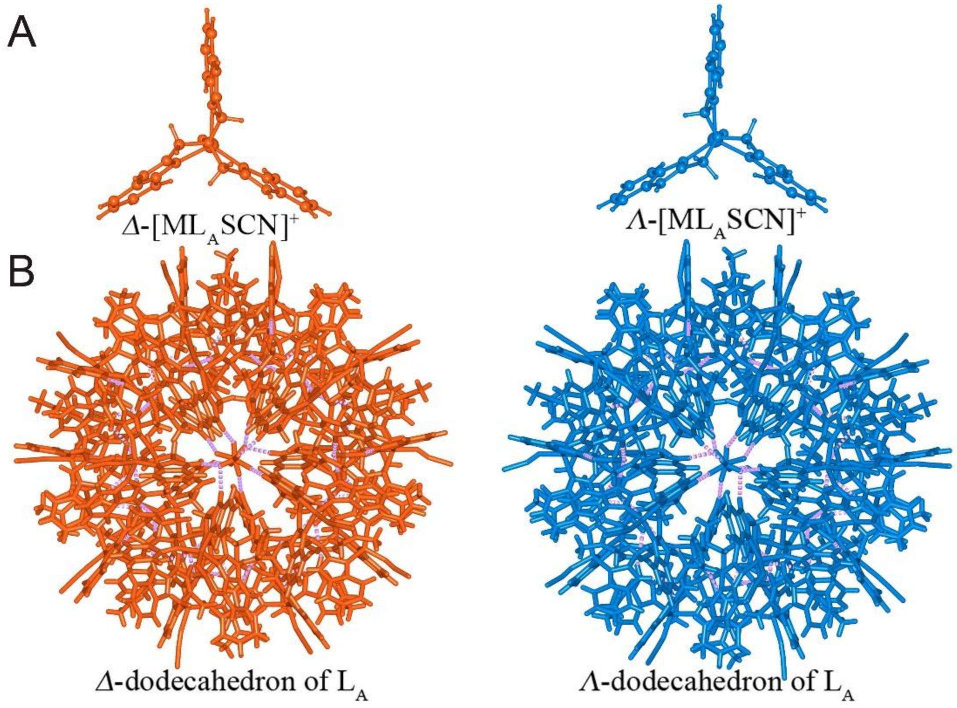

Fig. S6. Enantiomers of [ML$_A$SCN]$^+$ and a dodecahedron comprising [ML$_A$SCN]$^+$. (**A**) *Δ*- and *Λ*-[ML$_A$SCN]$^+$. (**B**) *Δ*- and *Λ*-dodecahedron comprising [ML$_A$SCN]$^+$.

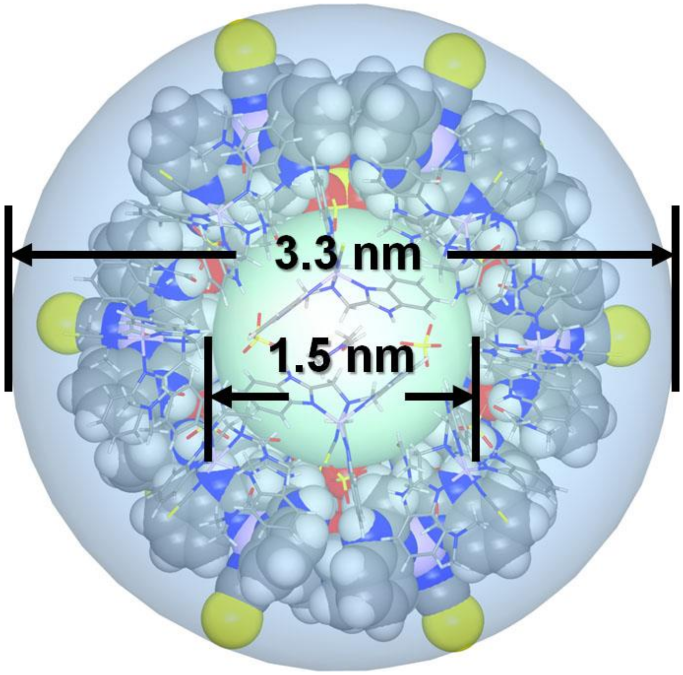

Fig. S7. Circumscribed and inscribed spheres defining the shells of **Zn-H-cage**.

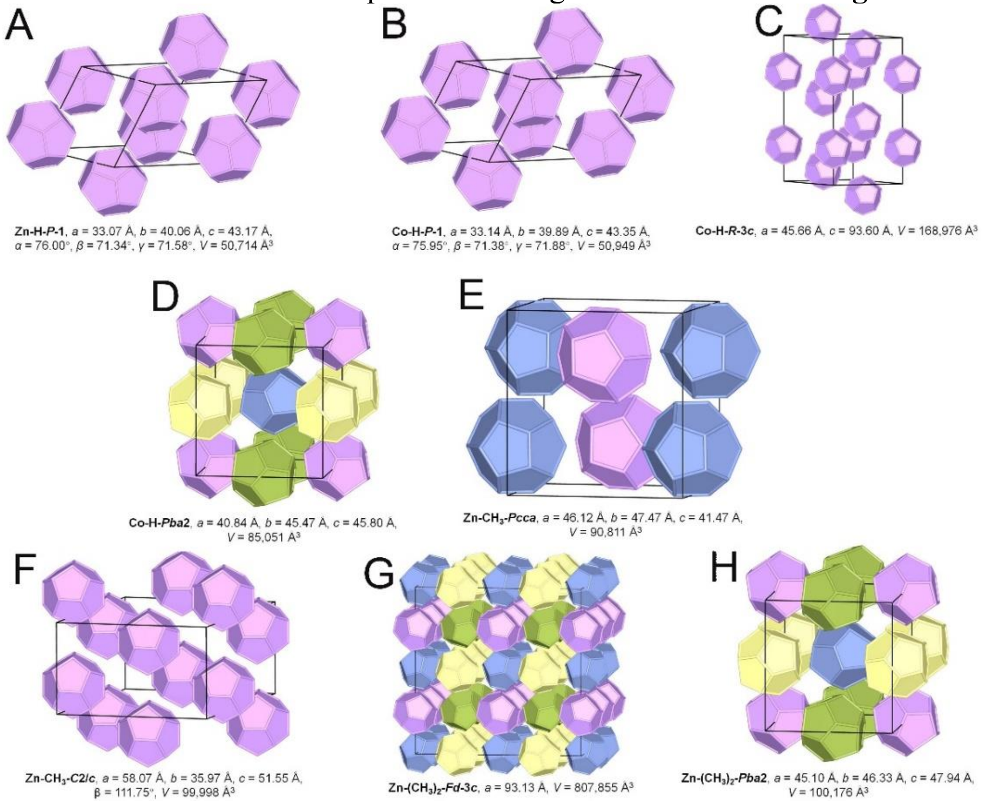

Fig. S8. Packings of spherical dodecahedra in the lattices of the obtained eight phases. (A) Packing of dodecahedra in **Zn-H-*P*–1**. (B) Packing of dodecahedra in **Co-H-*P*–1**. (C) Packing of dodecahedra in **Co-H-*R*–3*c***. (D) Packing of dodecahedra in **Co-H-*Pba*2**. (E) Packing of dodecahedra in **Zn-CH_3-*Pcca***. (F) Packing of dodecahedra in **Zn-CH_3-*C*2/*c***. (G) Packing of dodecahedra in **Zn-$(CH_3)_2$-*Fd*–3*c***. (H) Packing of dodecahedra in **Zn-$(CH_3)_2$- *Pba*2**.

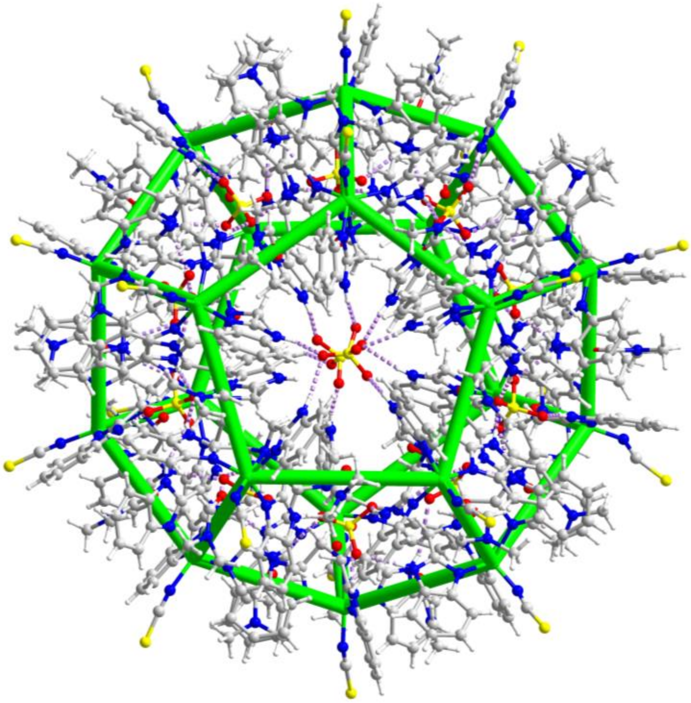

Fig. S9. Asymmetry unit of **Co-H-*P*–1** containing an intact **Co-H-cage**.

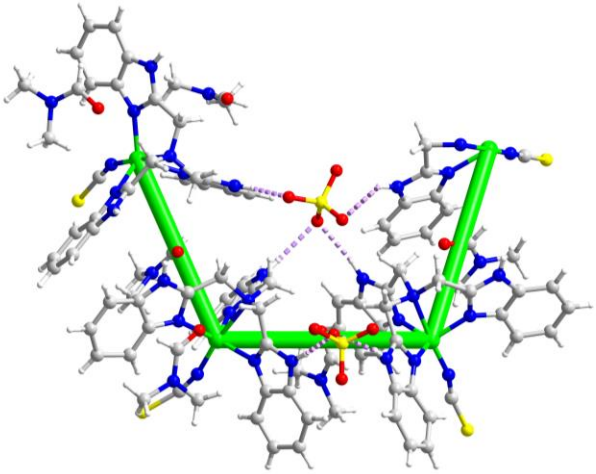

Fig. S10. Asymmetry unit of **Co-H-*R*–3*c*** containing 1/6 **Co-H-cage**.

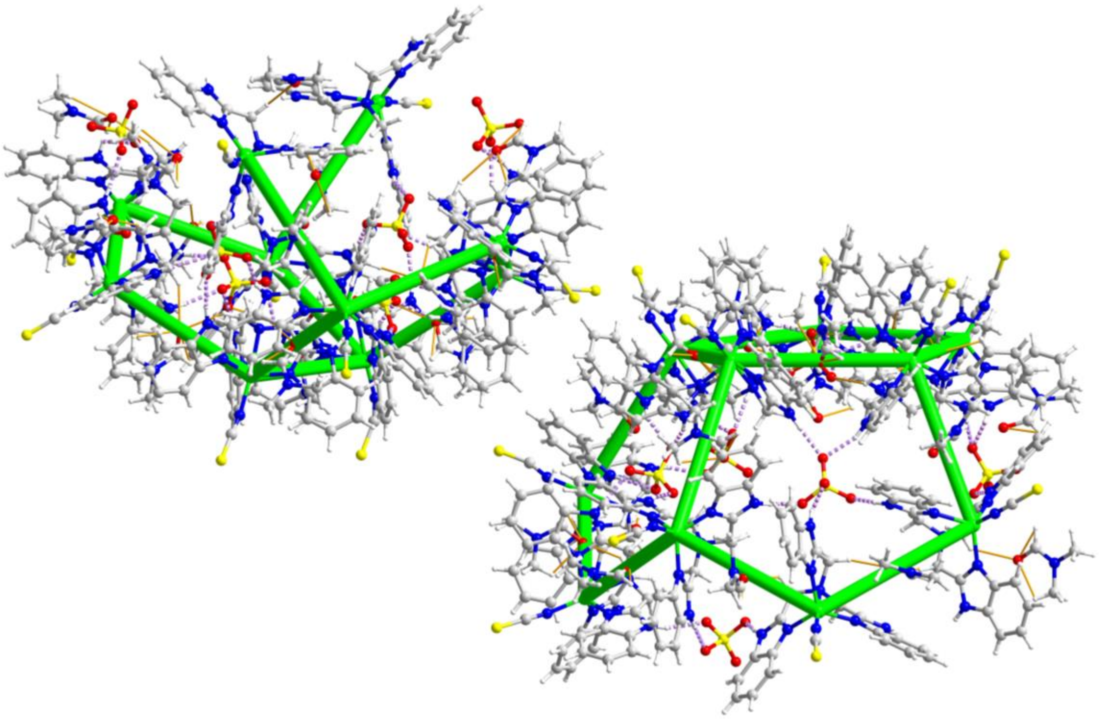

Fig. S11. Asymmetry unit of **Co-H-*Pba*2** containing two halves of **Co-H-cage**.

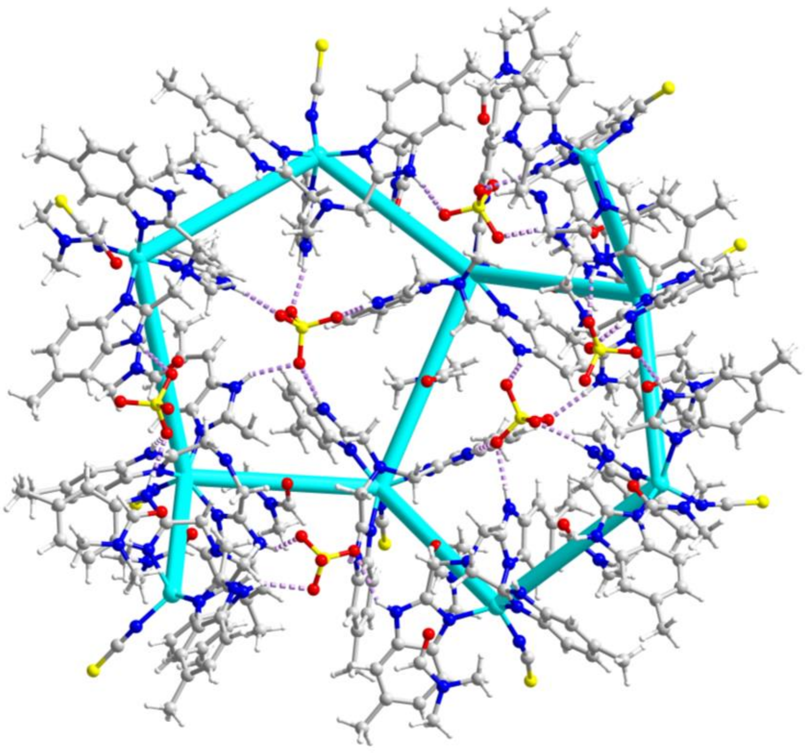

Fig. S12. Asymmetry unit of **Zn-CH₃-*Pcca*** containing 1/2 **Zn-CH₃-cage**.

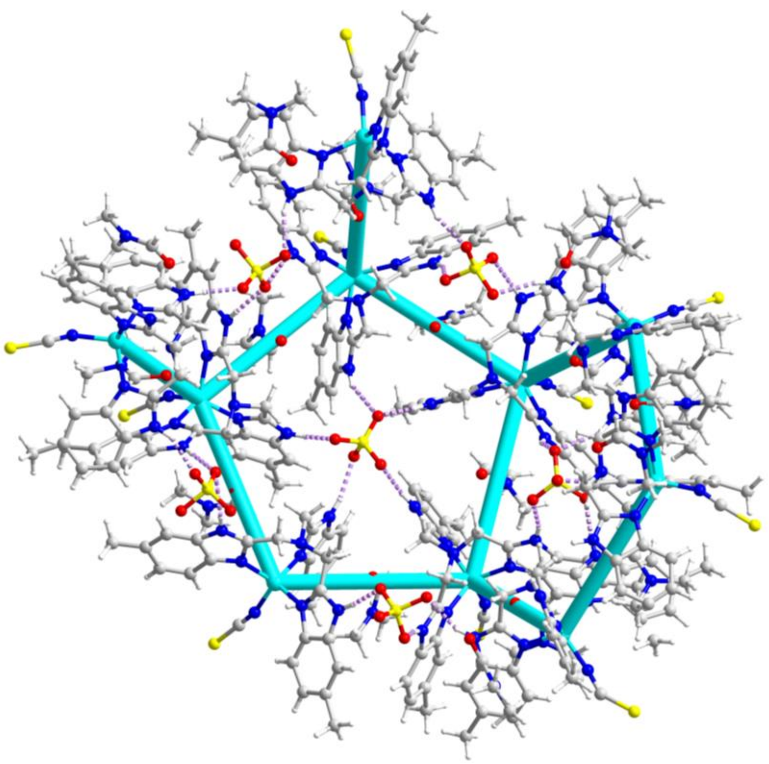

Fig. S13. Asymmetry unit of **Zn-CH$_3$-*C*2/*c*** containing 1/2 **Zn-CH$_3$-cage**.

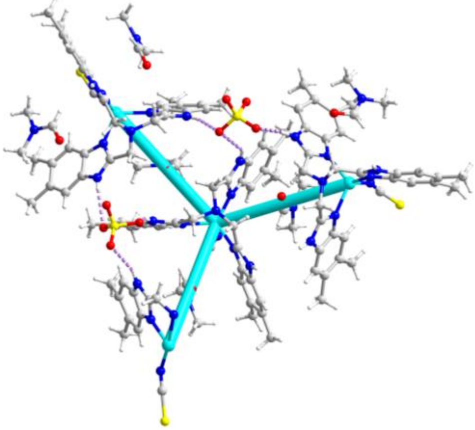

Fig. S14. Asymmetry unit of **Zn-(CH$_3$)$_2$-*Fd*–3*c*** containing 1/6 **Zn-(CH$_3$)$_2$-cage**.

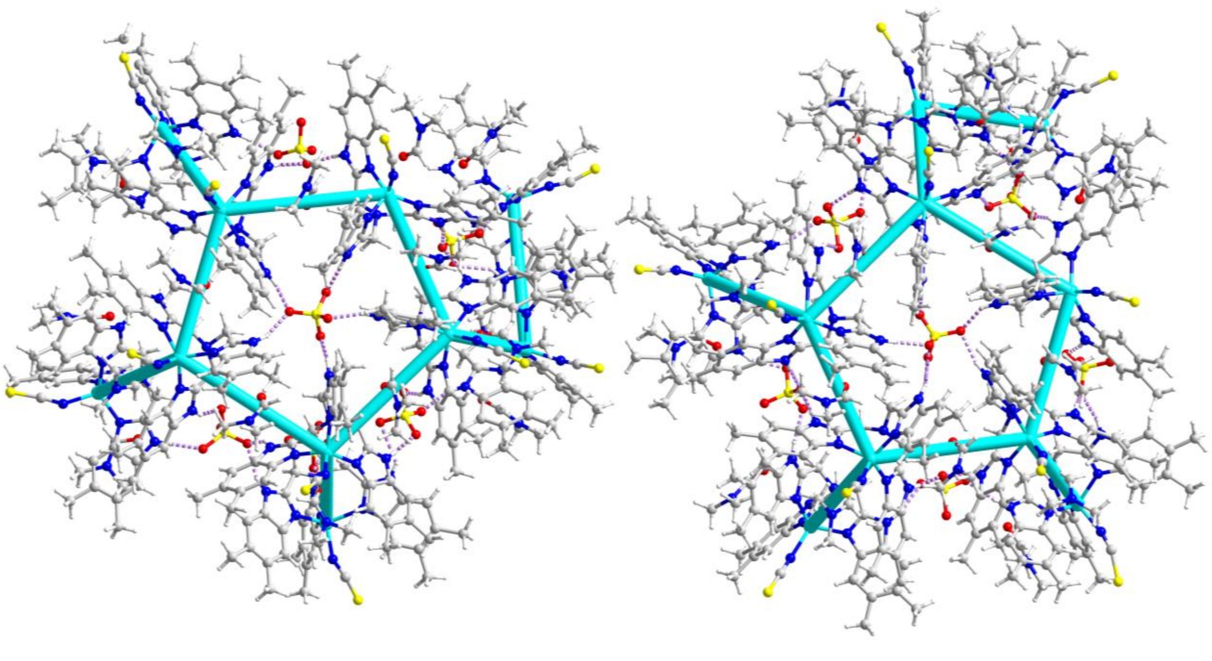

Fig. S15. Asymmetry unit of **Zn-$(CH_3)_2$-*Pba*2** containing two halves of **Zn-$(CH_3)_2$-cage**.

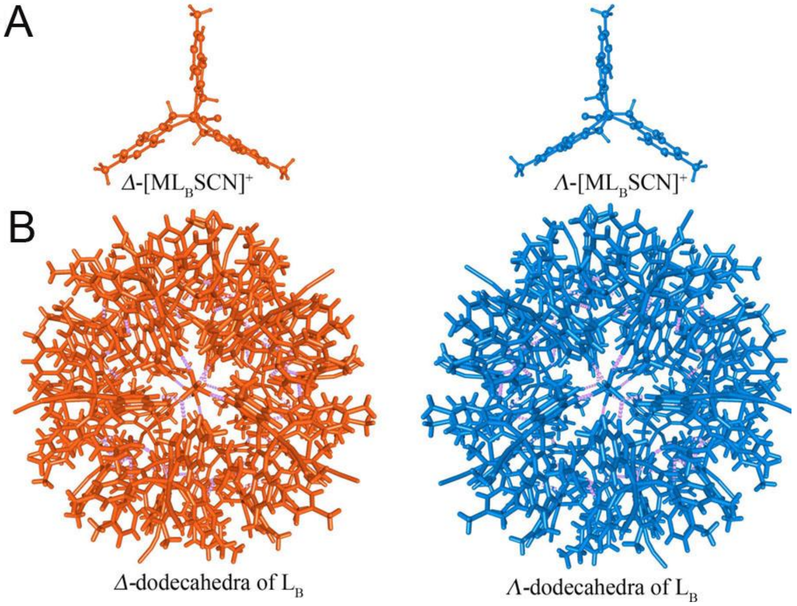

Fig. S16. Enantiomers of $[ML_BSCN]^+$ and dodecahedron comprising L_B. (**A**) Δ- and Λ-$[ML_BSCN]^+$. (**B**) Δ- and Λ-dodecahedron comprising L_B.

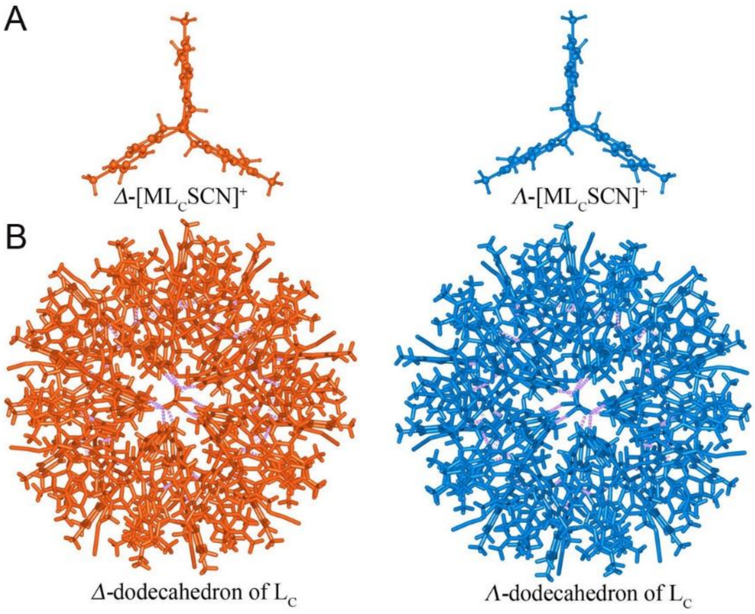

Fig. S17. Enantiomers of [ML$_C$SCN]$^+$ and dodecahedron comprising L$_C$. (**A**) *Δ*- and *Λ*-[ML$_C$SCN]$^+$. (**B**) *Δ*- and *Λ*-dodecahedra consisting of L$_C$.

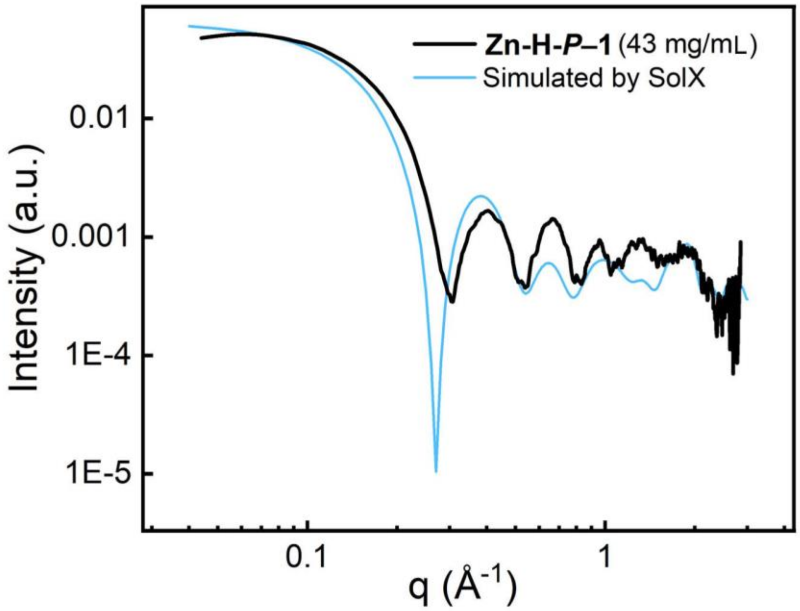

Fig. S18. Simulated and experimental SAXS patterns of 43 mg/mL **Zn-H-cage-*P*–1** in DMF.

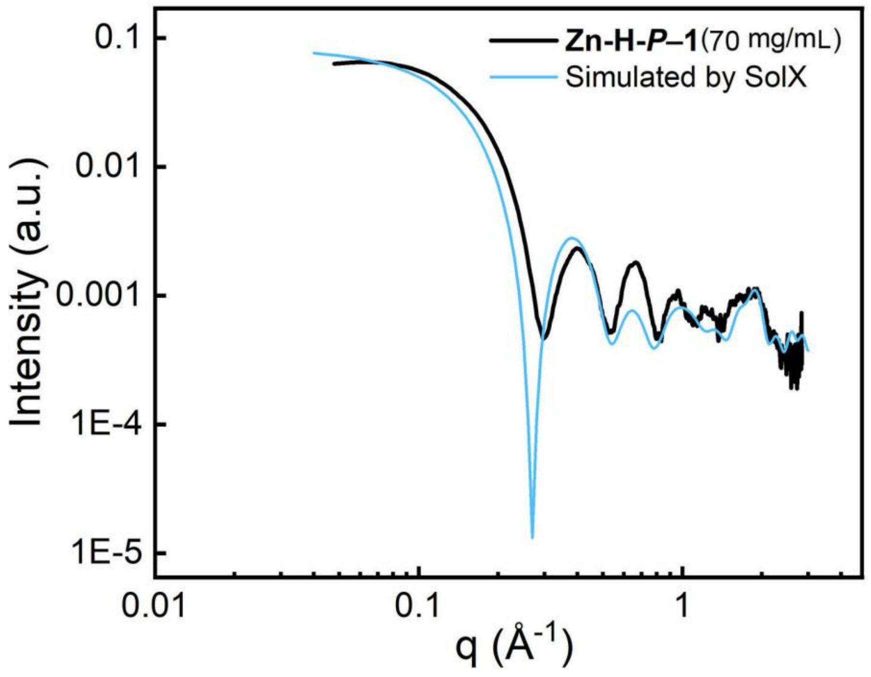

Fig. S19. Simulated and experimental SAXS patterns of 70 mg/mL **Zn-H-cage-*P*–1** in DMF.

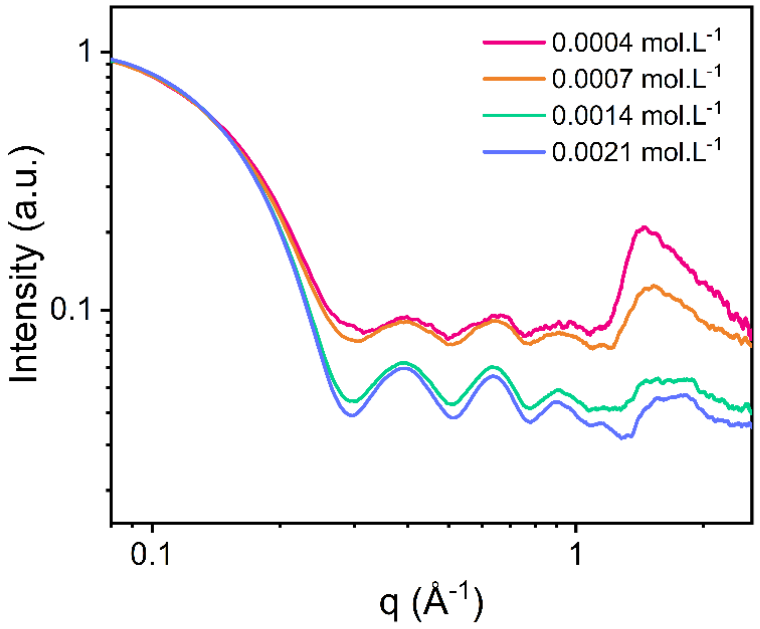

Fig. S20. Experimental SAXS patterns of **Zn-H-cage-*P*–1** in DMF over a series of concentrations.

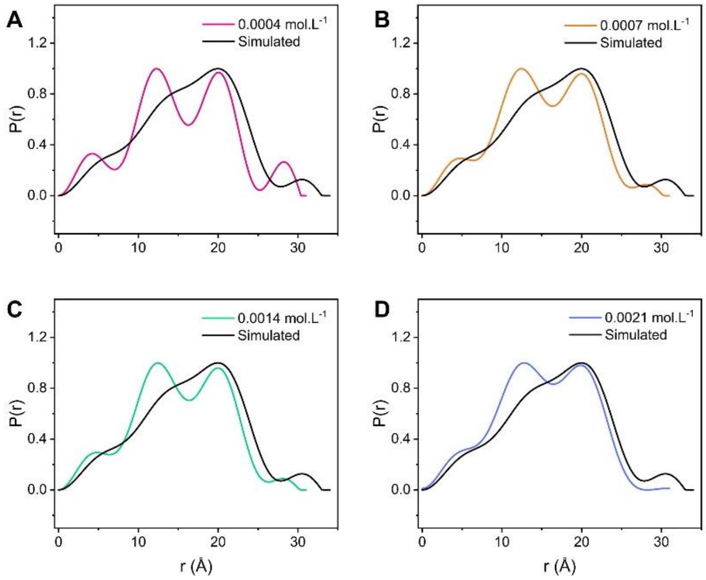

Fig. S21. PDDFs of different solutions of Zn-H-cage at different concentrations. (**A**) PDDF of the solution at 0.0004 mol·L^{-1}, (**B**) PDDF of the solution at 0.0007 mol·L^{-1}, (**C**) PDDF of the solution at 0.0017 mol·L^{-1}, and (**D**) PDDF of the solution at 0.0021 mol·L^{-1}.

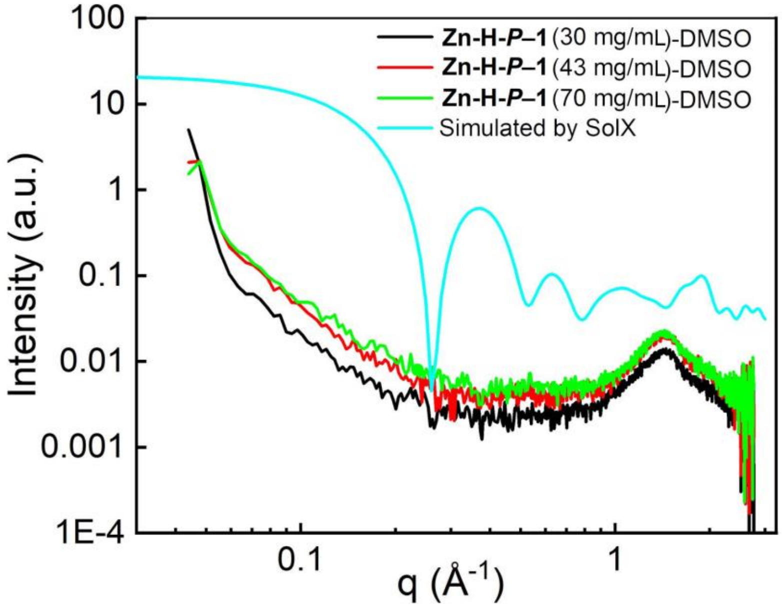

Fig. S22. Simulated and experimental SAXS patterns of **Zn-H-cage-*P*–1** in DMSO at different concentration condition.

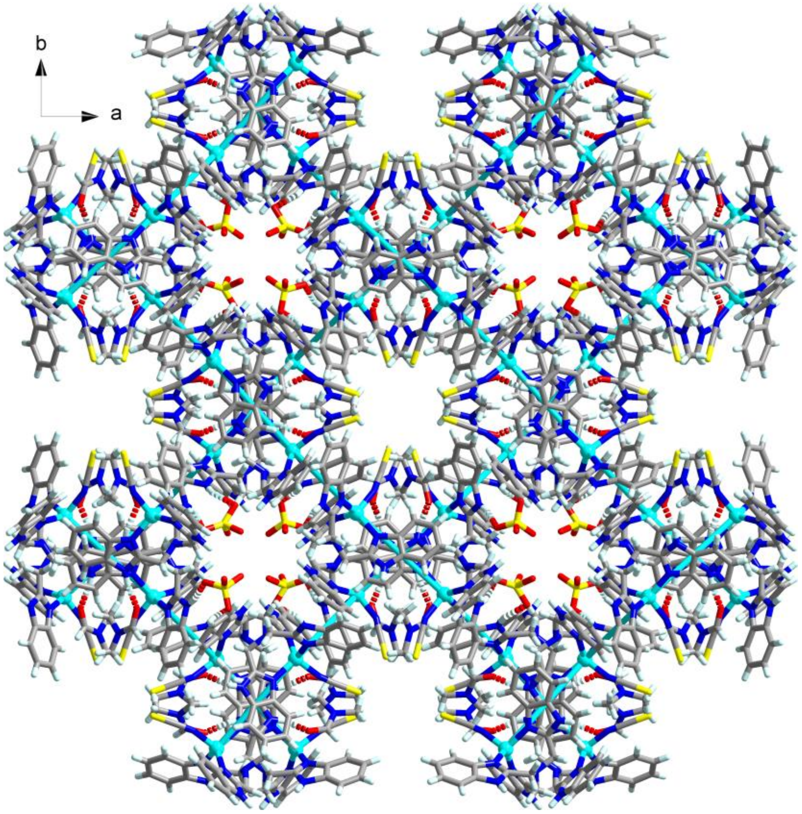

Fig. S23. Structure of **1** showing the channels along [0 1 1] direction.

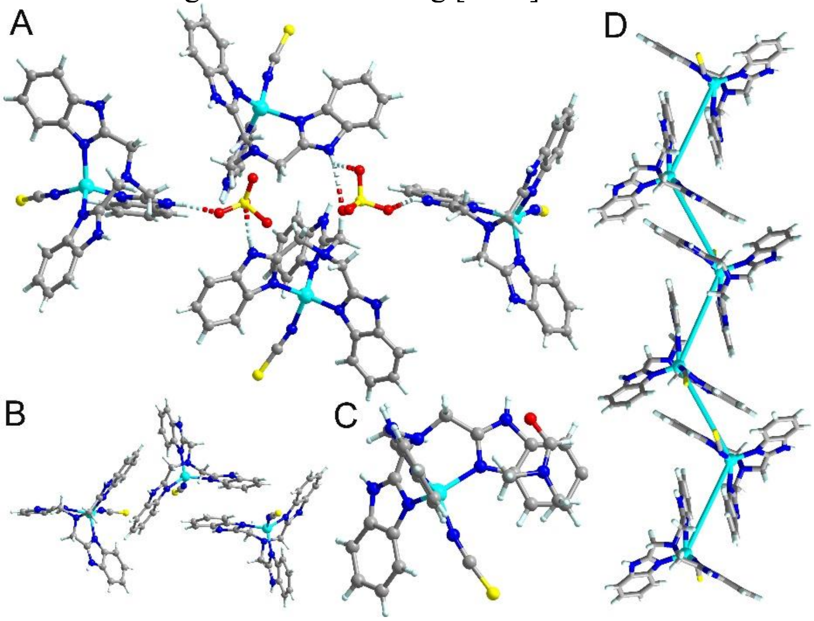

Fig. S24. Non-covalent interactions and π-stacked zigzag chain in 1. (**A**) Anion coordination. (**B**) $\pi \cdots \pi$ interaction between benzimidazoylmethyl-arms. (**C**) amide–π interaction between benzimidazoylmethyl-arm and DMF. (**D**) π-stacked zigzag chain of $[ZnLSCN]^{+}$ in **1**.

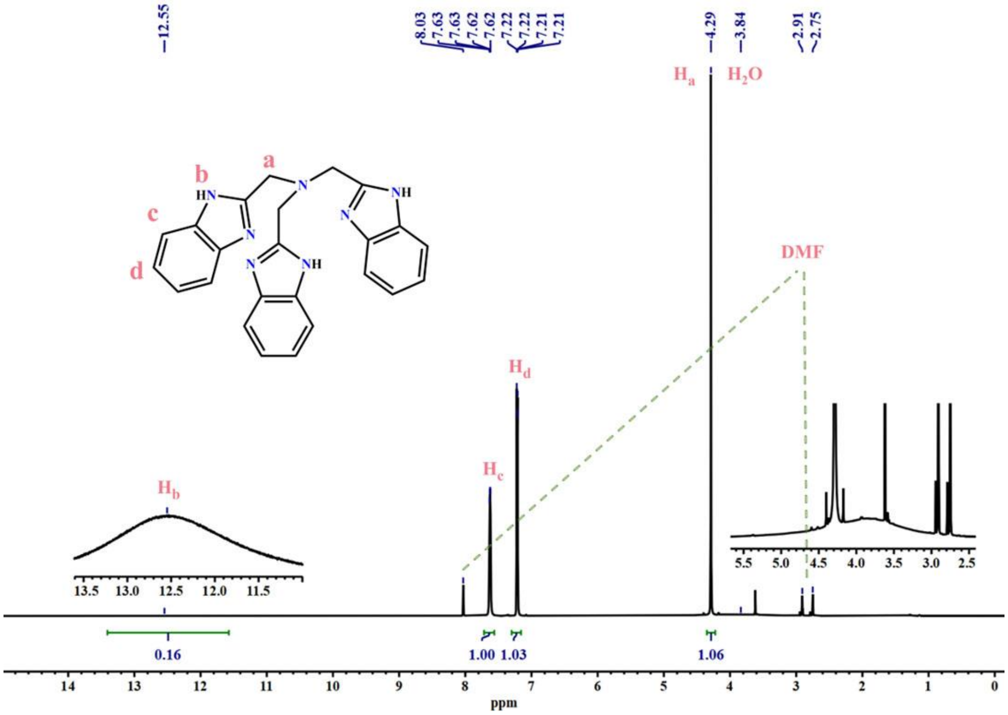

Fig. S25. ^{1}H NMR spectrum of L_A (600MHz, DMF-*d*7, 298K).

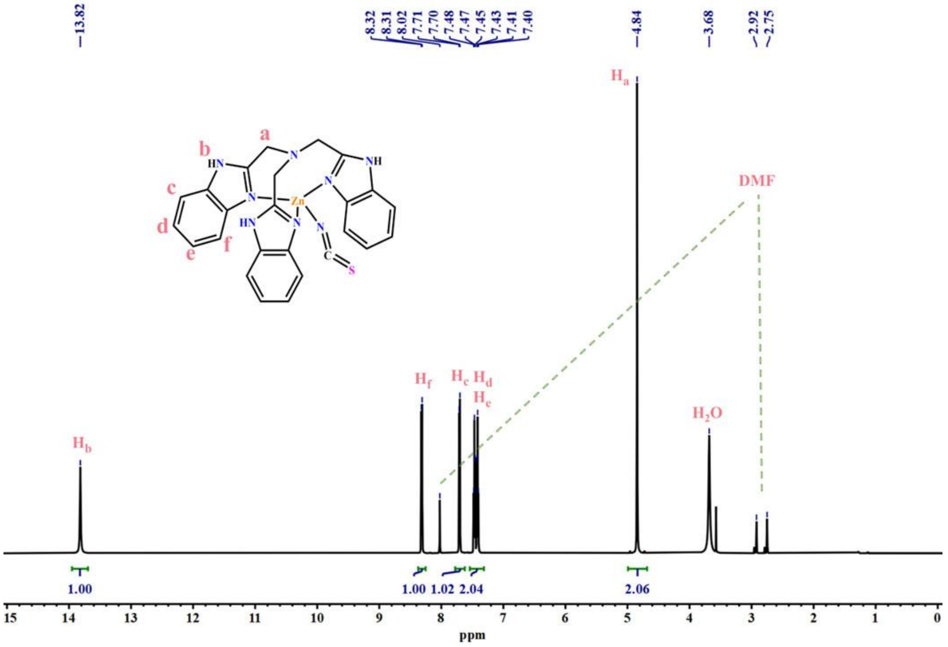

Fig. S26. ^{1}H NMR spectrum of the mixture (L_A+$Zn(SCN)_2$) (600MHz, DMF-*d*7, 298K).

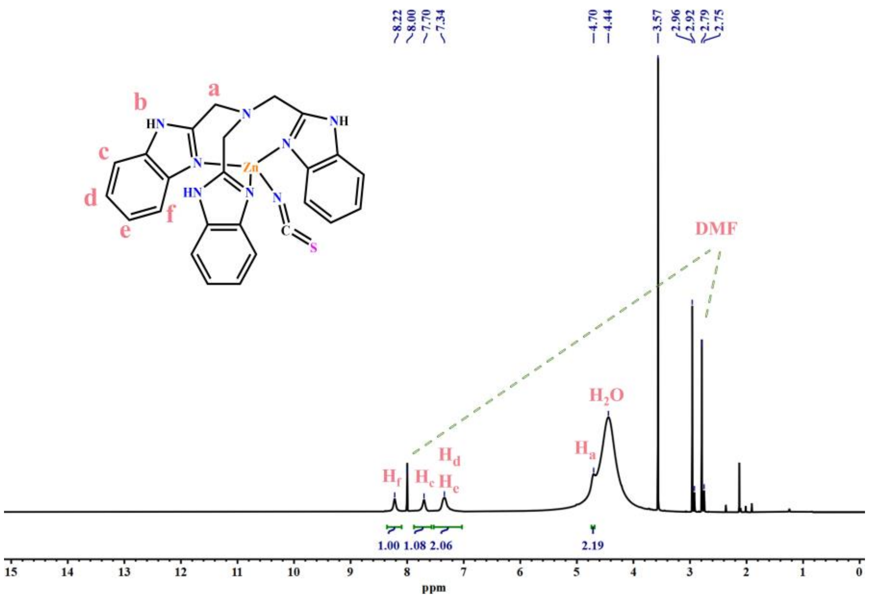

Fig. S27. ^{1}H NMR spectrum of the reacted solution after thermal treatment for synthesis of **Zn-H-*P*–1** (600MHz, DMF-*d*7, 298K).

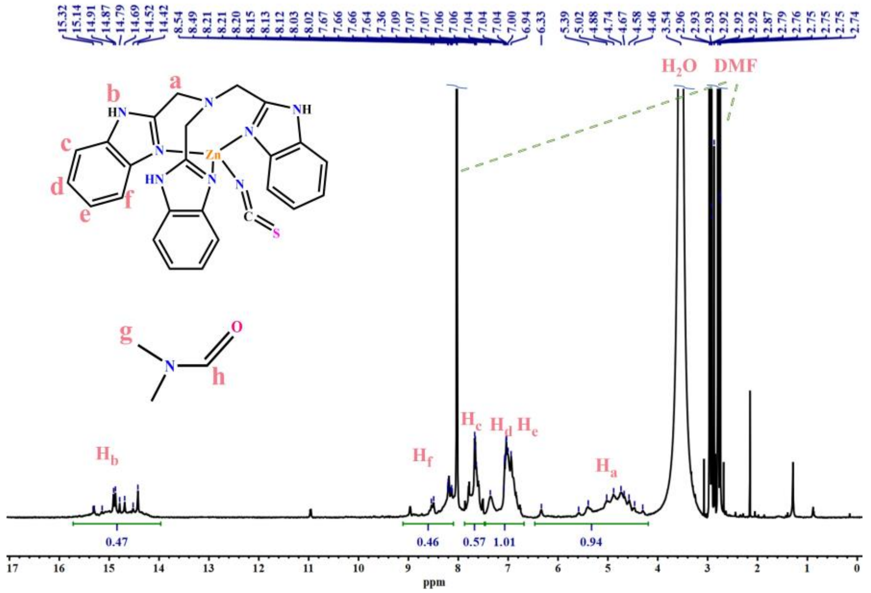

Fig. S28. ^{1}H NMR spectrum of the solution of **Zn-H-*P*–1** in DMF-*d*7 (600MHz, DMF-*d*7, 298K).

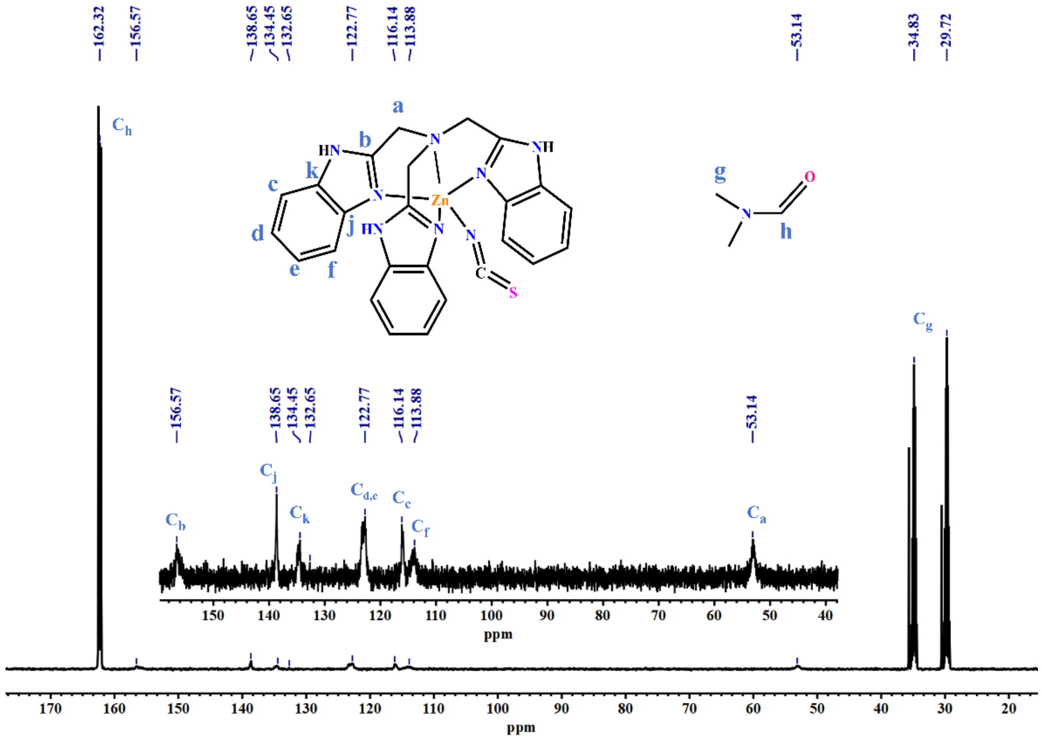

Fig. S29. ^{13}C NMR spectrum of the solution of **Zn-H-*P*–1** in DMF-*d*7 (600MHz, DMF-*d*7, 298K).

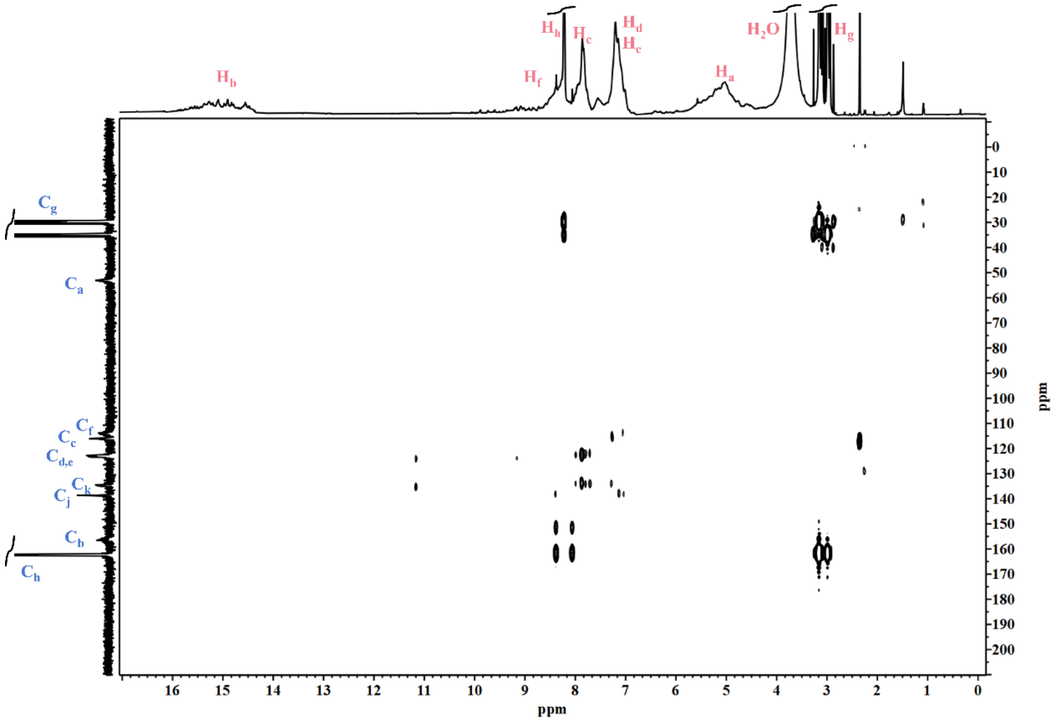

Fig. S30. ^{1}H–^{13}C HMBC NMR spectrum of the solution of **Zn-H-*P*–1** in DMF-*d*7 (600MHz, DMF-*d*7, 298K).

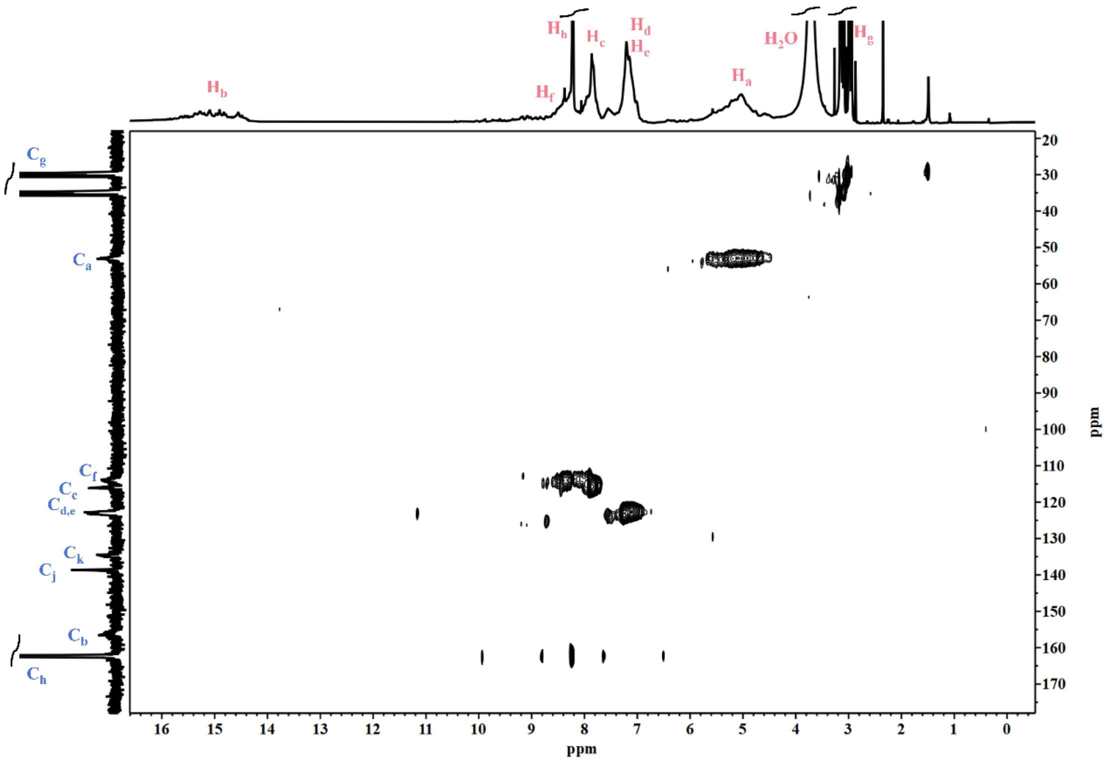

Fig. S31. ^{1}H–^{13}C HSQC NMR spectrum of the solution of **Zn-H-*P*–1** in DMF-*d*7 (600MHz, DMF-*d*7, 298K).

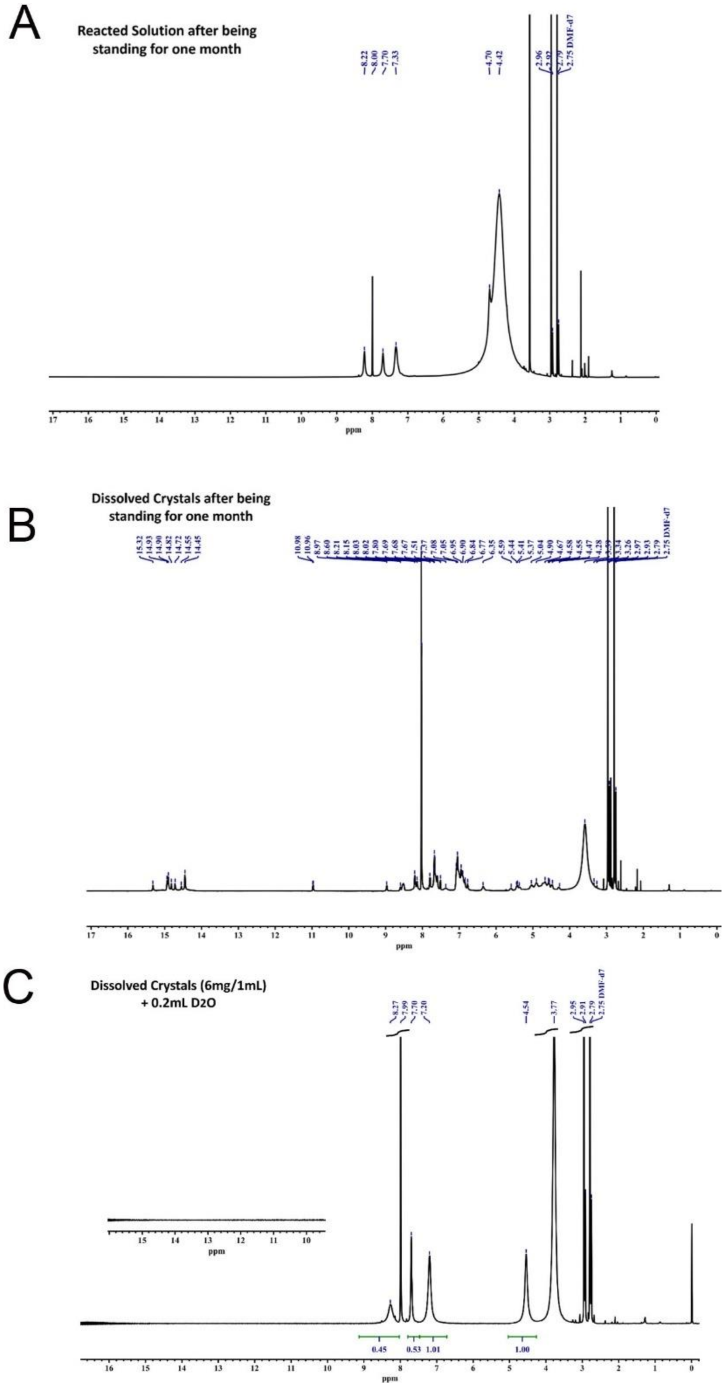

Fig. S32. ^{1}H NMR spectra. (**A**) ^{1}H NMR spectrum for the reacted solution after being kept undisturbed for one month. (**B**) ^{1}H NMR spectrum for the solution of dissolved crystals after being kept undisturbed for one month. (**C**) ^{1}H NMR spectrum for the solution of dissolved crystals after mixing with D_2O.

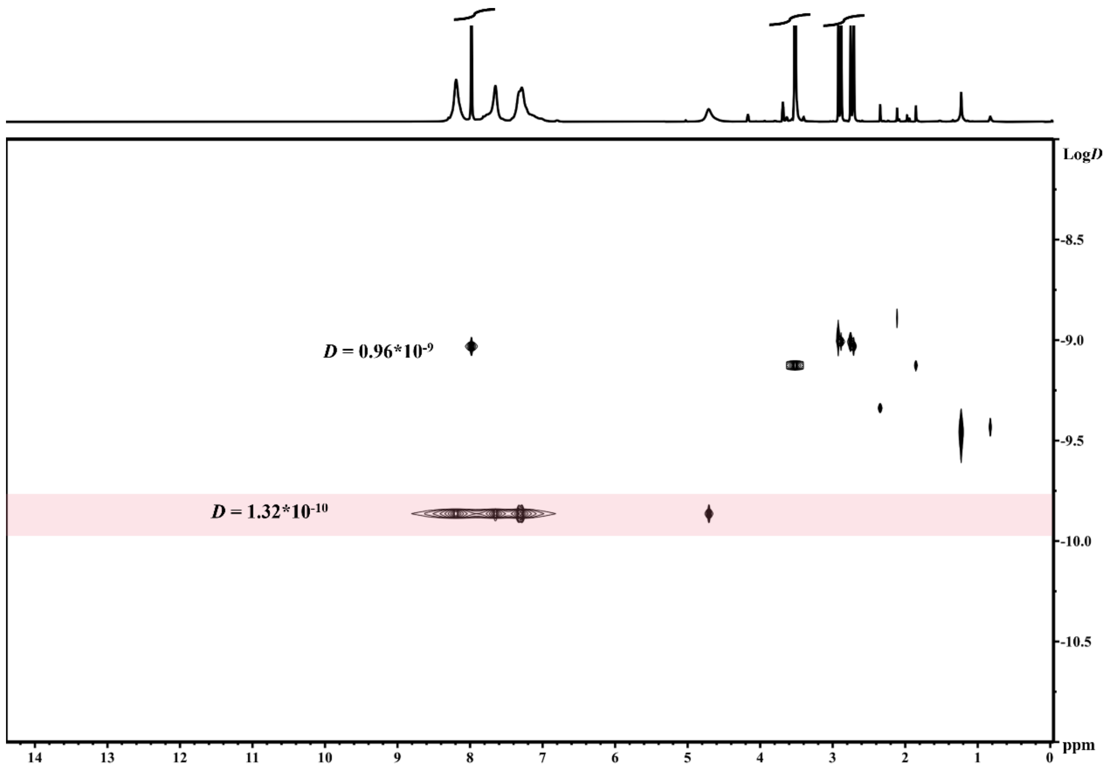

Fig. S33. ^{1}H DOSY spectrum of the reacted solution after thermal treatment for synthesis of **Zn-H-*P*–1** (600MHz, DMF-*d*7, 298K).

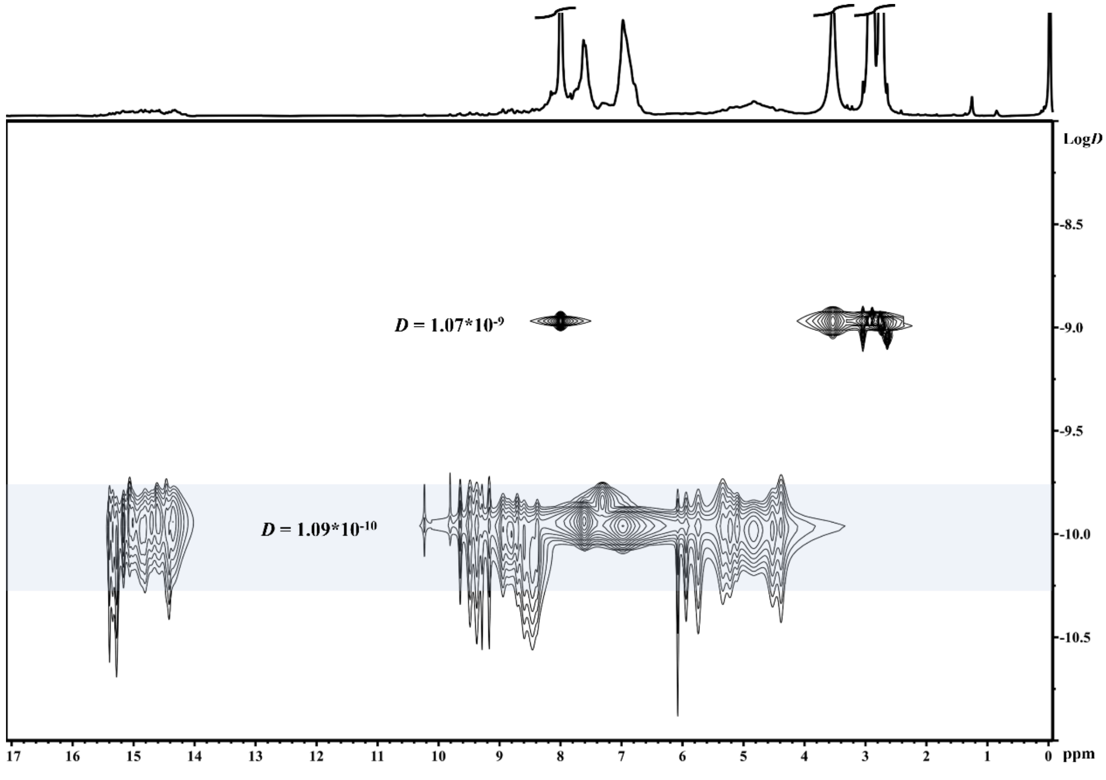

Fig. S34. ^{1}H DOSY spectrum of the solution of **Zn-H-*P*–1** in DMF-*d*7 (600MHz, DMF-*d*7, 298K).

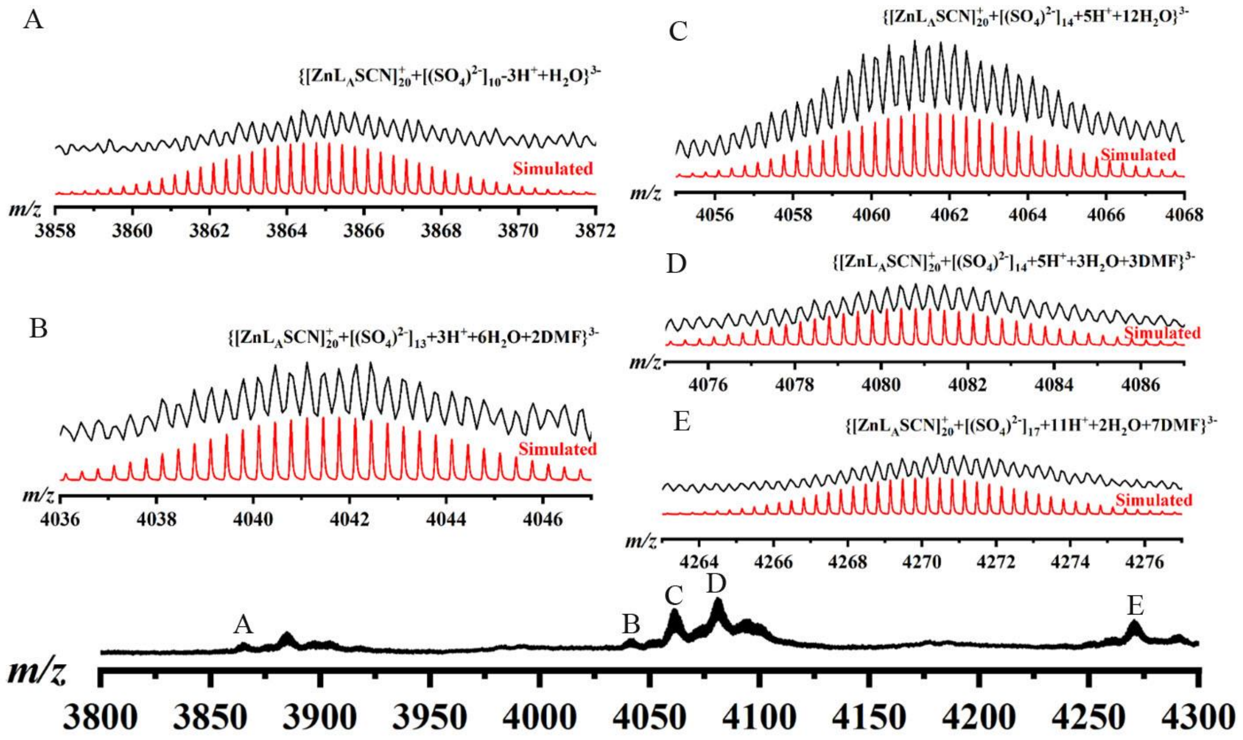

Fig. S35. HR-ESI-MS for the solution of **Zn-H-*P*–1** in DMF (m/z: 3800–4300). (**A**) HR-ESI-MS for the species of $\{[ZnL_ASCN]^+{}_{20}+[(SO_4)^{2-}]_{10}-3H^++H_2O\}^{3-}$. (**B**) HR-ESI-MS for the species of $\{[ZnL_ASCN]^+{}_{20}+[(SO_4)^{2-}]_{13}+3H^++6H_2O+2DMF\}^{3-}$. (**C**) HR-ESI-MS for the species of $\{[ZnL_ASCN]^+{}_{20}+[(SO_4)^{2-}]_{14}+5H^++12H_2O\}^{3-}$. (**D**) HR-ESI-MS for the species of $\{[ZnL_ASCN]^+{}_{20}+[(SO_4)^{2-}]_{14}+5H^++3H_2O+3DMF\}^{3-}$. (**E**) HR-ESI-MS for the species of $\{[ZnL_ASCN]^+{}_{20}+[(SO_4)^{2-}]_{17}+11H^++2H_2O+7DMF\}^{3-}$.

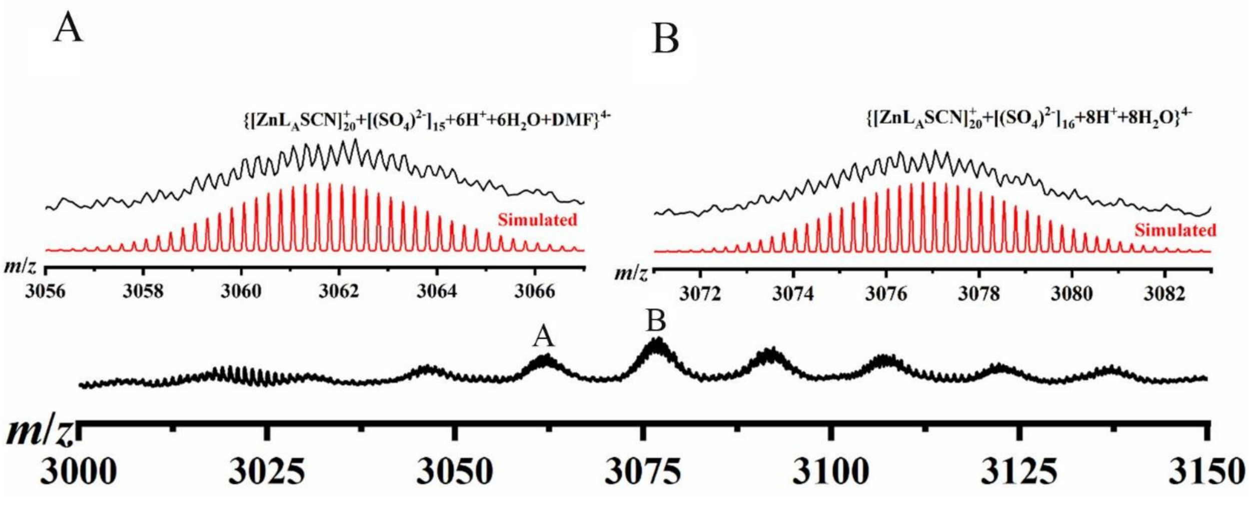

Fig. S36. HR-ESI-MS for the reacted solution for synthesizing **Zn-H-*P*–1**. (**A**) HR-ESI-MS for the species of $\{[ZnL_ASCN]^+{}_{20}+[(SO_4)^{2-}]_{15}+3H^++6H_2O+DMF\}^{4-}$. (**B**) HR-ESI-MS for the species of $\{[ZnL_ASCN]^+{}_{20}+[(SO_4)^{2-}]_{16}+8H^++8H_2O\ \}^{4-}$

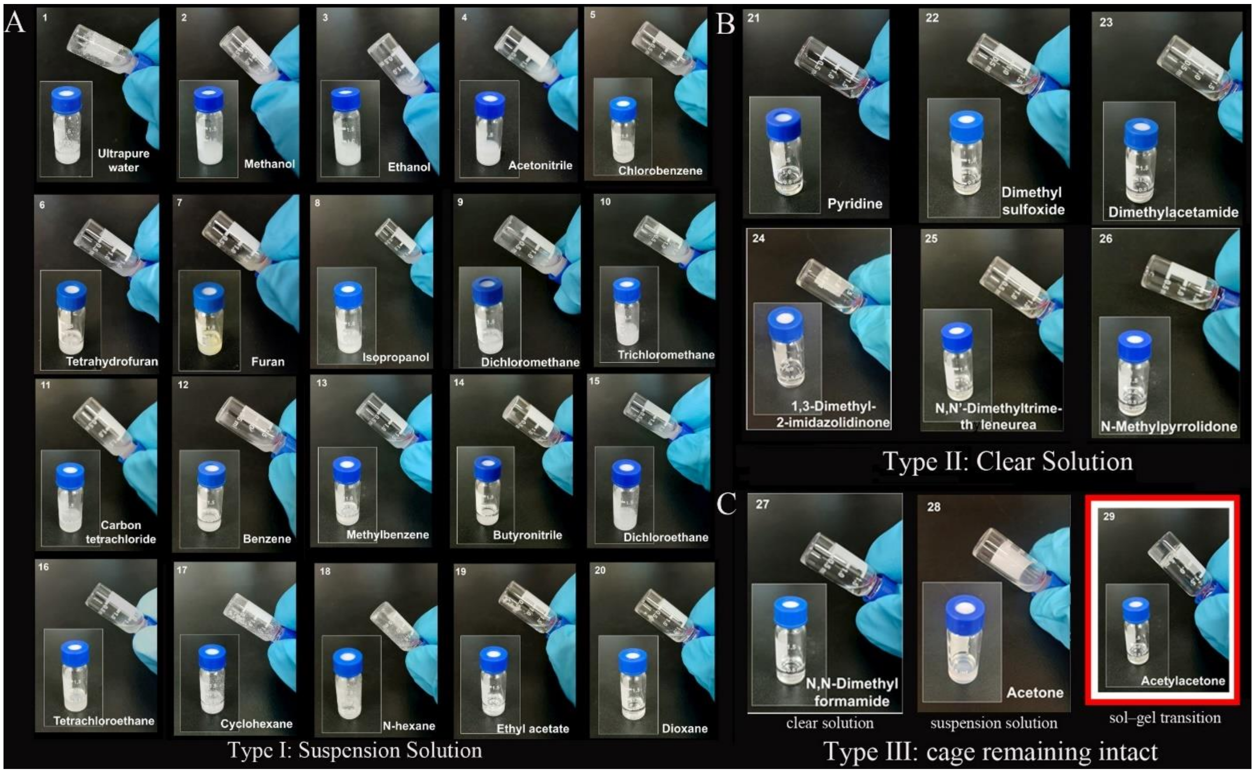

Fig. S37. Solubilities of Zn-H-*P*–1 in various solvents. Sonicating 5 mg **Zn-H-*P*–1** in 0.5 mL each of 29 different solvents leads to suspension solution (**A**) in 21 (1–20, 28) solvents and clear solution (**B**) in 8 solvents (21–26, 27, 29). **Zn-H-cage** may be stable (**C**) in DMF, acetone, and acac, but sol–gel transition was only observed in acac.

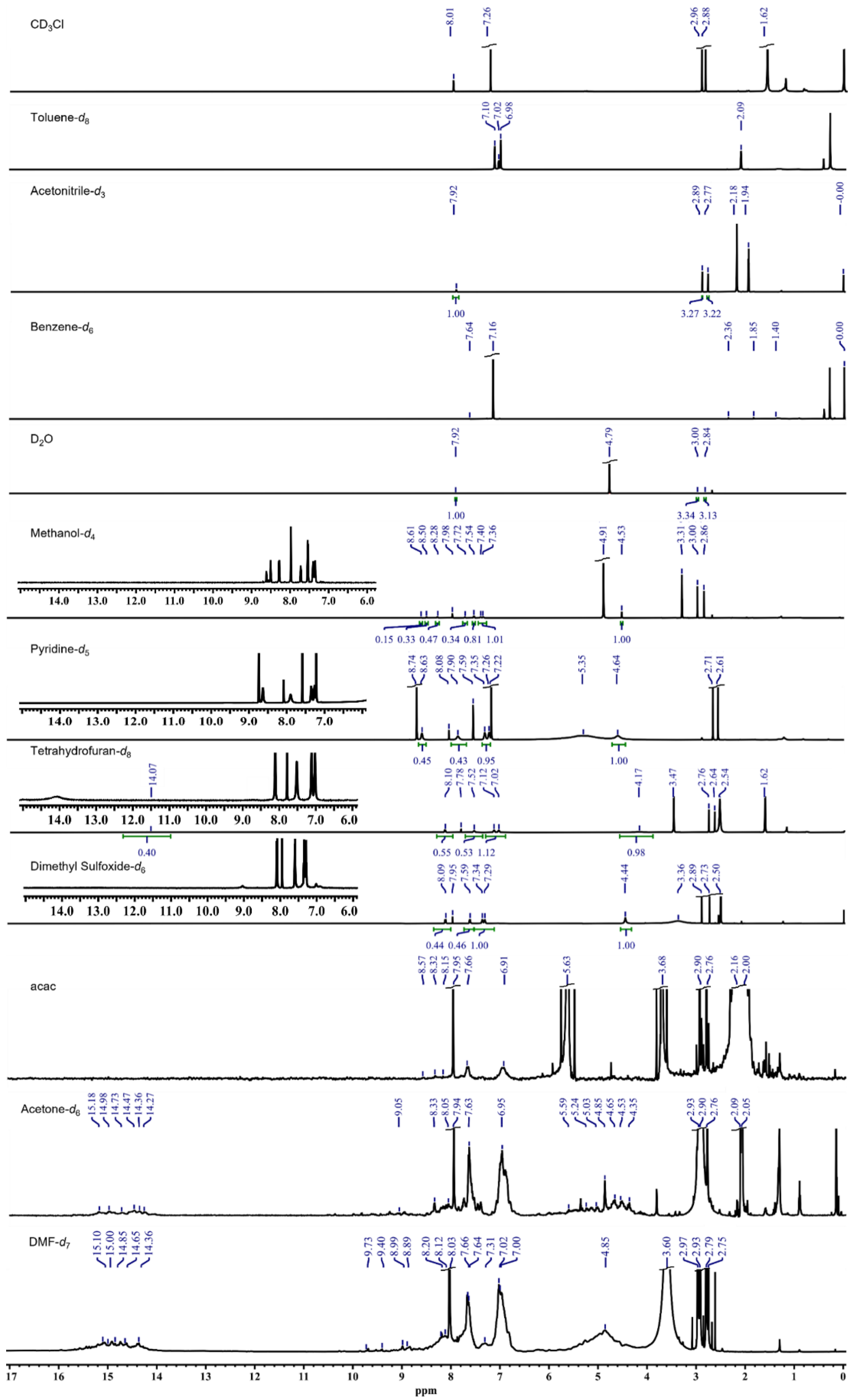

Fig. S38. ^{1}H NMR spectra of the solutions of crystals of **Zn-H-*P*–1** in different solvents. **Zn-H-cage** may be stable in DMF, acetone, and acac.

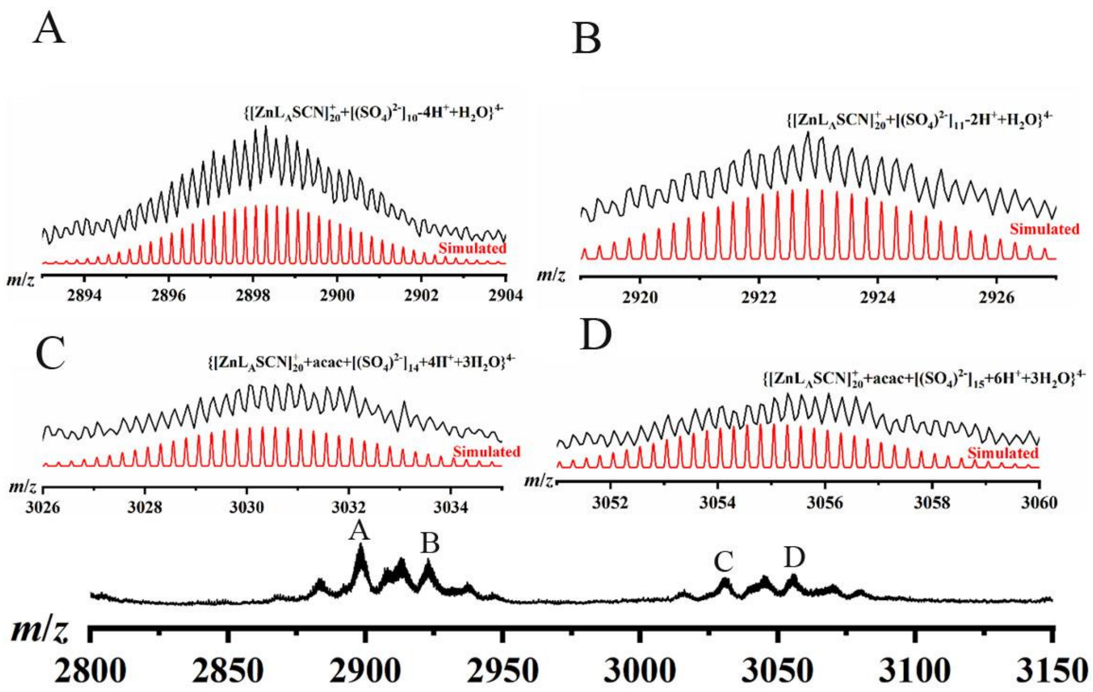

Fig. S39. HR-ESI-MS for the solution of Zn-H-*P*–1 in acac (m/z: 2800–3150). (**A**) HR-ESI-MS for the species of for the species of $\{[ZnL_ASCN]^+{}_{20}+[(SO_4)^{2-}]_{10}–4H^++H_2O\}^{4-}$. (**B**) HR-ESI-MS for the species of for the species of $\{[ZnL_ASCN]^+{}_{20}+[(SO_4)^{2-}]_{11}–2H^++H_2O\}^{4-}$. (**C**) HR-ESI-MS for the species of $\{[ZnL_ASCN]^+{}_{20}+acac+[(SO_4)^{2-}]_{14}+4H^++3H_2O\}^{4-}$. (D) HR-ESI-MS for the species of $\{[ZnL_ASCN]^+{}_{20}+acac+[(SO_4)^{2-}]_{15}+6H^++3H_2O\}^{4-}$.

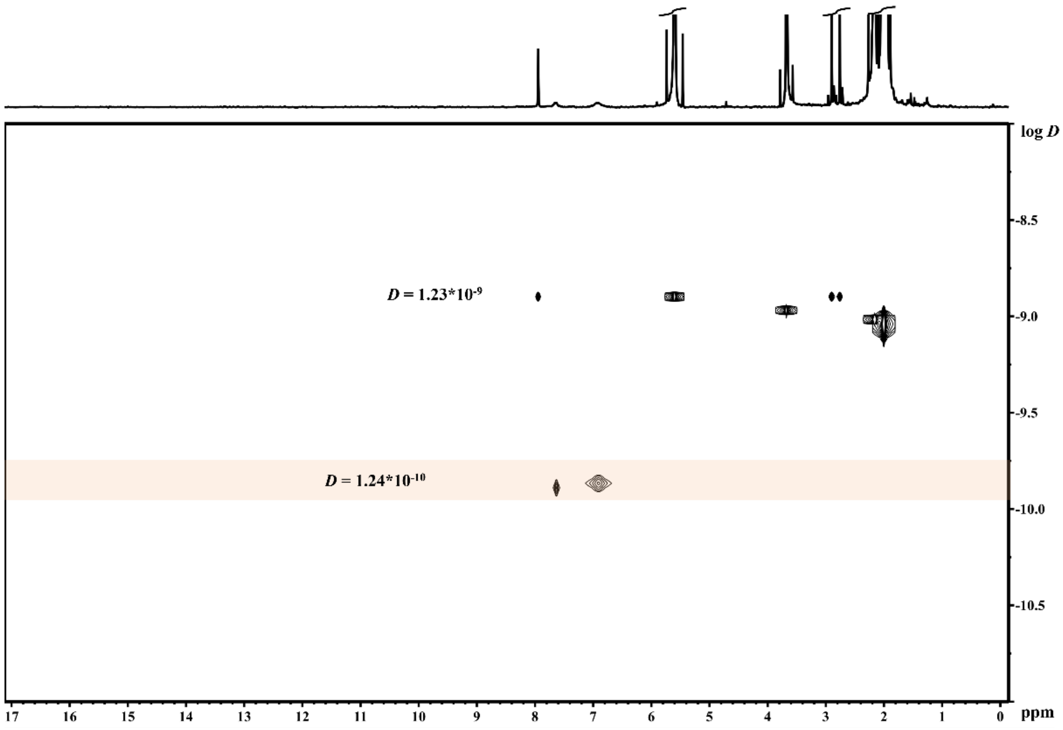

Fig. S40. ^{1}H NMR spectra for the solution of **Zn-H-*P*–1** in acac.

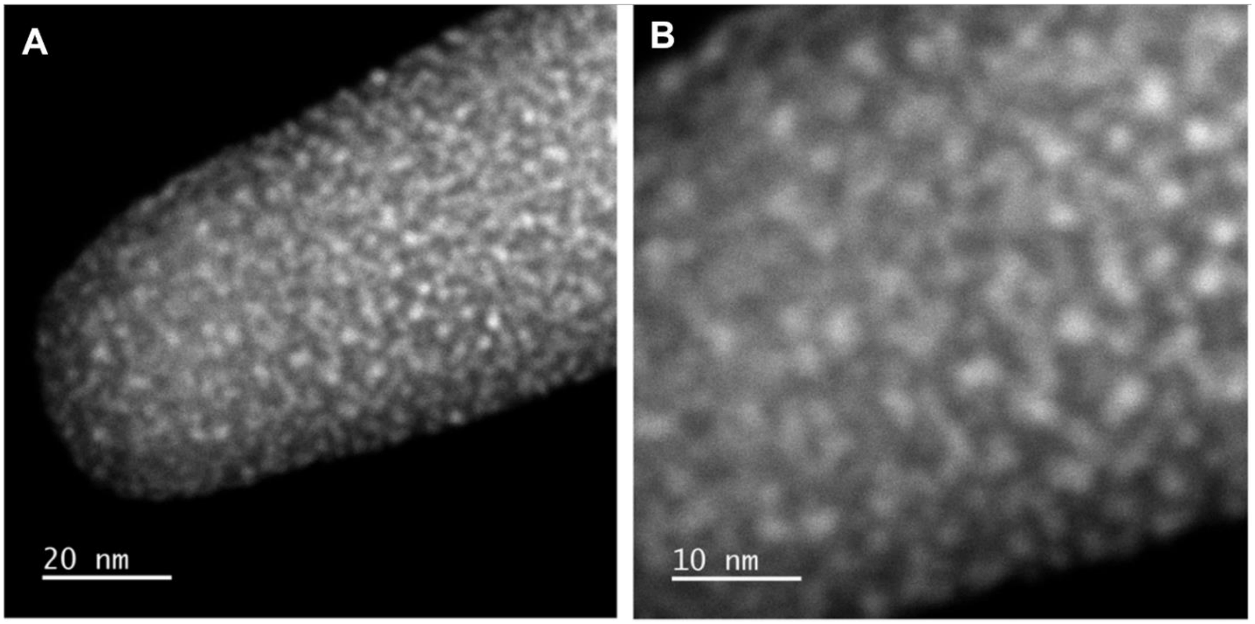

Fig. S41. TEM images of the gel of Zn-H-*P*–1 in acac. (**A**) TEM image with the scale bar of 20 nm. (**B**) TEM image with the scale bar of 10 nm.

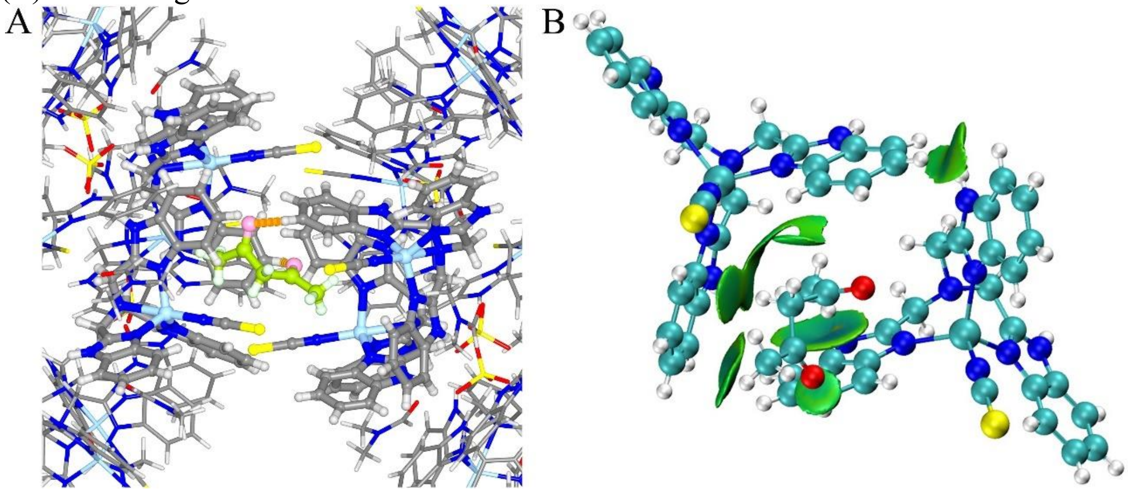

Fig. S42. Proposed structure of acac bridging two individual Zn-H-cages. (**A**) Model of one acac molecule bridging two individual **Zn-H-cages**. (**B**) Proposed sandwich-type π–π interaction between acac and benzimidazoylmethyl arm of **Zn-H-cage.**

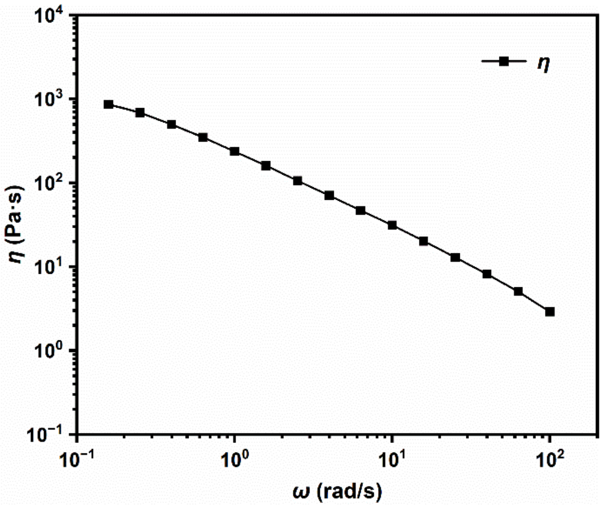

Fig. S43. Viscosity (η) of the gel on frequency sweep.

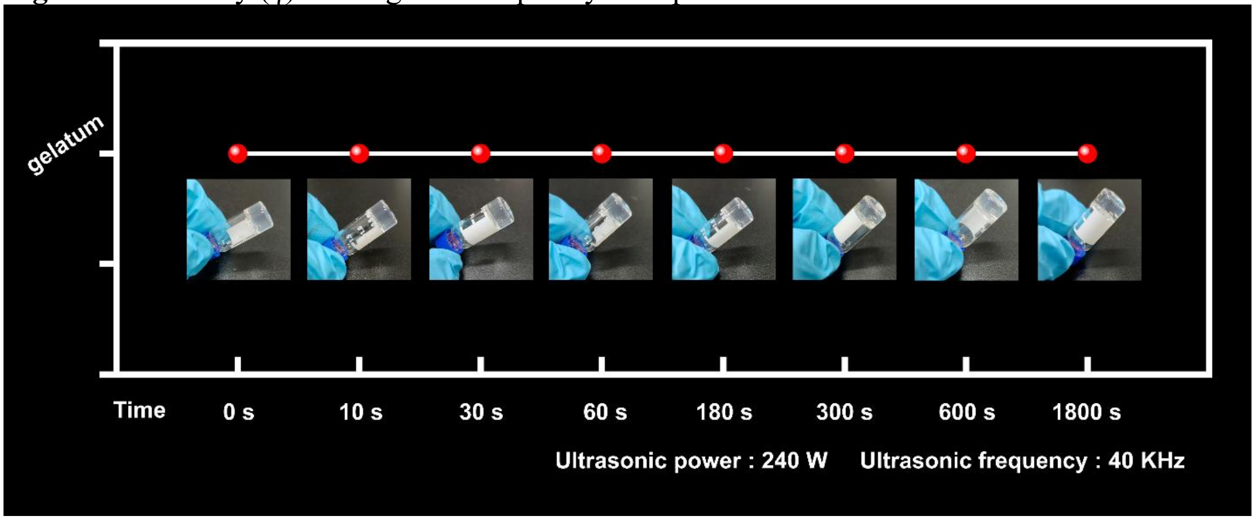

Fig. S44. Photographs showing the gel remains intact after sonication for 30 min.

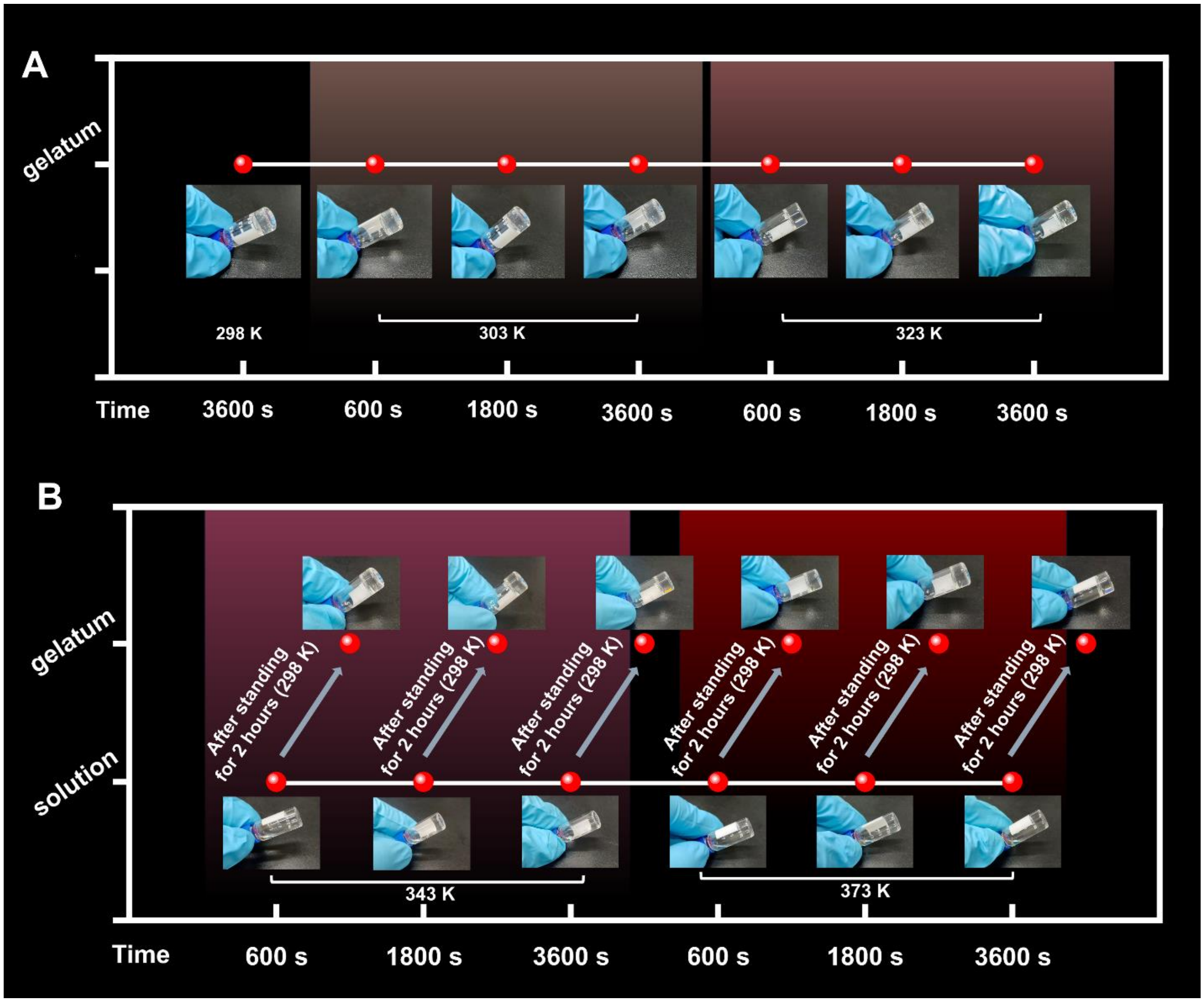

Fig. S45. Photographs showing the gel disassembled after heating at 70 ºC for 10 min. (**A**) Photographs showing the gel remained below 50 ºC. (**B**) Photographs showing the sol–gel upon heating and cooling.

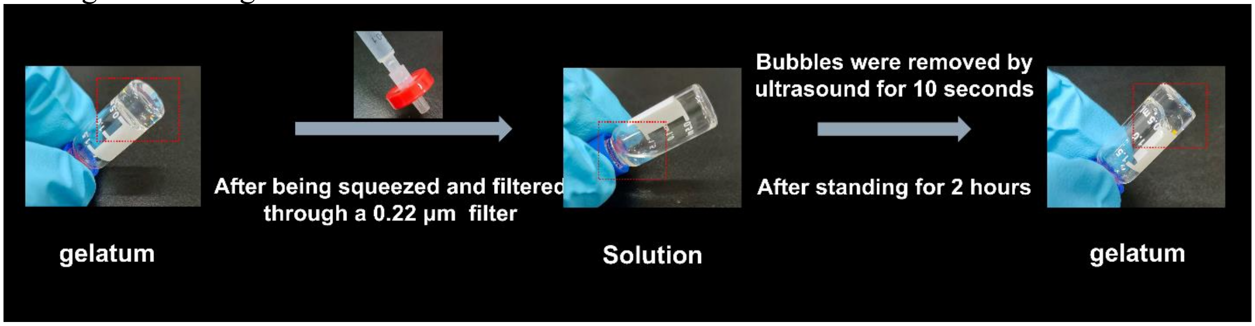

Fig. S46. Photographs showing the gel disassembled after being squeezed by plastic injector.

Table S1. Crystallographic data of **Zn-H-*P*–1**.

	**Zn-H-*P*–1**
Formula	$C_{590}H_{630}N_{190}O_{78}S_{32}Zn_{20}$
Formula weight	13964.12
Temp. (*K*)	99.99(10)
Crystal System	Triclinic
Space group	$P\bar{1}$
a (Å)	33.0707(4)
b (Å)	40.0583(5)
c (Å)	43.1654(5)
α (°)	76.0050(10)
β (°)	71.1380(10)
γ (°)	71.5750(10)
V (Å^3)	50714.0(12)
Z	2
$\rho_{cal.}$ (g cm^{-3})	0.914
μ	1.557
F (000)	14472.0
2*θ range* (°)	4.096 to 135.42
Reflections ($I > 2\theta$)	681737
R_1 ($I > 2\theta$)	0.0962
wR_2 (*all*)	0.3604
GOF on F^2	1.029
CCDC#	2456179

Table S2. Crystallographic data of **Co-H-*P*–1**.

	**Co-H-*P*–1**
Formula	$C_{590}H_{630}N_{190}O_{78}S_{32}Co_{20}$
Formula weight	13835.32
Temp. (*K*)	100.00(14)
Crystal System	Triclinic
Space group	$P\bar{1}$
a (Å)	33.1423(12)
b (Å)	39.8854(14)
c (Å)	43.3461(14)
α (°)	75.947(3)
β (°)	71.381(3)
γ (°)	71.883(3)
V (Å^3)	50949(3)
Z	2
$\rho_{cal.}$ (g cm^{-3})	0.902
μ	3.521
F (000)	14352.0
2*θ range* (°)	4.186 to 120
Reflections ($I > 2\theta$)	420836
R_1 ($I > 2\theta$)	0.1141
wR_2 (*all*)	0.3501
GOF on F^2	0.797
CCDC#	2456055

Table S3. Crystallographic data of **Co-H-*R*–3*c***.

	**Co-H-*R*–3*c***
Formula	$C_{590}H_{630}N_{190}O_{78}S_{32}Co_{20}$
Formula weight	13835.32
Temp. (*K*)	100(2)
Crystal System	Trigonal
Space group	$R\bar{3}c$
a (Å)	45.657(3)
c (Å)	93.601(5)
V (Å^3)	168974(22)
Z	6
$\rho_{cal.}$ (g cm^{-3})	0.816
μ	3.185
F (000)	43056.0
2θ *range* (°)	4.388 to 97.324
Reflections ($I > 2\theta$)	39301
R_1 ($I > 2\theta$)	0.1375
wR_2 (*all*)	0.4679
GOF on F^2	0.907
CCDC#	2456059

Table S4. Crystallographic data of **Co-H-*Pba*2**.

	**Co-H-*Pba*2**
Formula	$C_{590}H_{630}N_{190}O_{78}S_{32}Co_{20}$
Formula weight	13835.32
Temp. (*K*)	99.99(11)
Crystal System	Orthorhombic
Space group	*Pba*2
a (Å)	40.8383(2)
b (Å)	45.4688(2)
c (Å)	45.8033(2)
V (Å^3)	85050.7(7)
Z	4
$\rho_{cal.}$ (g cm^{-3})	1.080
μ	4.219
F (000)	28704.0
2θ *range* (°)	4.328 to 148.07
Reflections ($I > 2\theta$)	2057199
R_1 ($I > 2\theta$)	0.0878
wR_2 (*all*)	0.2642
GOF on F^2	1.057
CCDC#	2456058

Table S5. Crystallographic data of **Zn-CH$_3$-*Pcca***.

	**Zn-CH$_3$-*Pcca***
Formula	$C_{650}H_{750}N_{190}O_{78}S_{32}Zn_{20}$
Formula weight	14805.68
Temp. (*K*)	100.00(10)
Crystal System	Orthorhombic
Space group	*Pcca*
a (Å)	46.1231(6)
b (Å)	47.4731(7)
c (Å)	41.4735(4)
V (Å^3)	90811(2)
Z	4
$\rho_{cal.}$ (g cm^{-3})	1.083
μ	1.763
F (000)	30864.0
2θ *range* (°)	4.65 to 140
Reflections ($I > 2\theta$)	328753
R_1 ($I > 2\theta$)	0.1148
wR_2 (*all*)	0.3790
GOF on F^2	1.190
CCDC#	2456066

Table S6. Crystallographic data of **Zn-CH$_3$-*C*2/*c***.

	**Zn-CH$_3$-*C*2/*c***
Formula	$C_{650}H_{750}N_{190}O_{78}S_{32}Zn_{20}$
Formula weight	14805.68
Temp. (*K*)	100.00(10)
Crystal System	Monoclinic
Space group	*C*2/*c*
a (Å)	58.0660(6)
b (Å)	35.9669(3)
c (Å)	51.5499(6)
β (°)	111.7460(10)
V (Å^3)	99998.0(19)
Z	4
$\rho_{cal.}$ (g cm^{-3})	0.983
μ	1.601
F (000)	30864.0
2θ *range* (°)	4.226 to 147.73
Reflections ($I > 2\theta$)	1101448
R_1 ($I > 2\theta$)	0.1073
wR_2 (*all*)	0.3827
GOF on F^2	1.133
CCDC#	2456065

Table S7. Crystallographic data of **Zn- $(CH_3)_2$-*Fd*–3*c***.

	**Zn-$(CH_3)_2$-*Fd*–3*c***
Formula	$C_{710}H_{870}N_{190}O_{78}S_{32}Zn_{20}$
Formula weight	15651.61
Temp. (*K*)	100.01(10)
Crystal System	Cubic
Space group	$Fd\bar{3}c$
a (Å)	93.1346(9)
V (Å^3)	807854(23)
Z	32
$\rho_{cal.}$ (g cm^{-3})	1.029
μ	1.608
F (000)	262272.0
2θ *range* (°)	5.614 to 100.856
Reflections ($I > 2\theta$)	184322
R_1 ($I > 2\theta$)	0.2100
wR_2 (*all*)	0.5677
GOF on F^2	1.761
CCDC#	2456062

Table S8. Crystallographic data of **Zn-$(CH_3)_2$-*Pba*2**.

	**Zn-$(CH_3)_2$-*Pba*2**
Formula	$C_{710}H_{870}N_{190}O_{78}S_{32}Zn_{20}$
Formula weight	15647.23
Temp. (*K*)	100.00(10)
Crystal System	Orthorhombic
Space group	*Pba*2
a (Å)	45.0999(6)
b (Å)	46.3292(6)
c (Å)	47.9441(7)
V (Å^3)	100176(2)
Z	4
$\rho_{cal.}$ (g cm^{-3})	1.037
μ	1.620
F (000)	32784.0
2θ *range* (°)	4.288 to 157.35
Reflections ($I > 2\theta$)	416077
R_1 ($I > 2\theta$)	0.0558
wR_2 (*all*)	0.1658
GOF on F^2	0.973
CCDC#	2456063

Table S9. Crystallographic data of **1**.

	**1**
Formula	$C_{112}H_{112}N_{36}O_{17}S_6Zn_4$
Formula weight	2698.29
Temp. (*K*)	100.00(10)
Crystal System	Orthorhombic
Space group	*Iba*2
a (Å)	37.234(5)
b (Å)	37.918(9)
c (Å)	17.588(2)
V (Å^3)	24831(7)
Z	8
$\rho_{cal.}$ (g cm^{-3})	1.444
μ	0.942
F (000)	11184.0
2θ *range* (°)	3.968 to 55.146
Reflections ($I > 2\theta$)	242918
R_1 ($I > 2\theta$)	0.1051
wR_2 (*all*)	0.2876
GOF on F^2	1.076
CCDC#	2456060

Table S10. Comparison of Rg values obtained from different analysis methods

Sample	Rg from PDDF analysis* (Å)	Rg from Guinier analysis (Å)
0.0004 $mol \cdot L^{-1}$	11.48	11.65
0.0007 $mol \cdot L^{-1}$	11.30	12.03
0.0014 $mol \cdot L^{-1}$	11.29	11.97
0.0021 $mol \cdot L^{-1}$	11.30	12.09

* the PDDF analysis was conducted in the q range from 0.06 to 0.85 $Å^{-1}$.

REFERENCES

1. J. M. Grimes, J. N. Burroughs, P. Gouet, J. M. Diprose, S. Ziéntara, P. P. C. Mertens, D. I. Stuart, The atomic structure of the bluetongue virus core. *Nature* **395**, 470–478 (1998).

2. X. H. Dai, Z. H. Zhou, Structure of the herpes simplex virus 1 capsid with associated tegument protein complexes. *Science* **360**, eaao7298 (2018).

3. T. F. Liu, Y. Liu, W. M. Xuan, Y. Cui, Chiral nanoscale metal-organic tetrahedral cages: Diastereoselective self-assembly and enantioselective separation. *Angew. Chem. Int. Ed. Engl. Engl.* **49**, 4121–4124 (2010).

4. D. Fujita, Y. Ueda, S. Sato, N. Mizuno, T. Kumasaka, M. Fujita, Self-assembly of tetravalent Goldberg polyhedra from 144 small components. *Nature* **540**, 563–566 (2016).

5. R. Chakrabarty, P. S. Mukherjee, P. J. Stang, Supramolecular coordination: Self-assembly of finite two- and three-dimensional ensembles. *Chem. Rev.* **111**, 6810–6918 (2011).

6. J. T. A. Jones, T. Hasell, X. F. Wu, J. Bacsa, K. E. Jelfs, M. Schmidtmann, S. Y. Chong, D. J. Adams, A. Trewin, F. Schiffman, F. Cora, B. Slater, A. Steiner, G. M. Day, A. I. Cooper, Modular and predictable assembly of porous organic molecular crystals. *Nature* **474**, 367–371 (2011).

7. K. Z. Su, W. J. Wang, S. F. Du, C. Q. Ji, M. Zhou, D. Q. Yuan, Reticular chemistry in the construction of porous organic cages. *J. Am. Chem. Soc.* **142**, 18060–18072 (2020).

8. P. Ballester, M. Fujita, J. Rebek Jr., Molecular containers. *Chem. Soc. Rev.* **44**, 392–393 (2015).

9. T. R. Cook, P. J. Stang, Recent developments in the preparation and chemistry of metallacycles and metallacages via coordination. *Chem. Rev.* **115**, 7001–7045 (2015).

10. Y. P. He, G. H. Chen, D. J. Li, Q. H. Li, L. Zhang, J. Zhang, Combining a titanium-organic cage and a hydrogen-bonded organic cage for highly effective third-order nonlinear optics. *Angew. Chem. Int. Ed. Engl.* **60**, 2920–2923 (2021).

11. W. Zuo, Y. Tao, Z. P. Luo, A. Y. Li, S. S. Wang, X. R. Qiao, F. Ma, C. D. Jia, Stereoselective assembly of hydrogen-bonded anionic cages dictated by organophosphate-based chiral nodes. *Angew. Chem. Int. Ed. Engl.* **62**, anie.202300470 (2023).

12. T. Sawada, Y. Inomata, K. Shimokawa, M. Fujita, A metal-peptide capsule by multiple ring threading. *Nat. Commun.* **10**, 5687 (2019).

13. M. D. Pluth, R. G. Bergman, K. N. Raymond, Acid catalysis in basic solution: A supramolecular host promotes orthoformate hydrolysis. *Science* **316**, 85–88 (2007).

14. G. Li, W. B. Yu, J. Ni, T. F. Liu, Y. Liu, E. H. Sheng, Y. Cui, Self-assembly of a homochiral nanoscale metallacycle from a metallosalen complex for enantioselective separation. *Angew. Chem. Int. Ed. Engl.* **47**, 1245–1249 (2008).

15. P. Mal, B. Breiner, K. Rissanen, J. R. Nitschke, White phosphorus is air-stable within a self-assembled tetrahedral capsule. *Science* **324**, 1697–1699 (2009).

16. S. Datta, M. L. Saha, P. J. Stang, Hierarchical assemblies of supramolecular coordination complexes. *Acc. Chem. Res.* **51**, 2047–2063 (2018).

17. Q. F. Sun, J. Iwasa, D. Ogawa, Y. Ishido, S. Sato, T. Ozeki, Y. Sei, K. Yamaguchi, M. Fujita, Self-assembled $M_{24}L_{48}$ polyhedra and their sharp structural switch upon subtle ligand variation. *Science* **328**, 1144–1147 (2010).

18. D. Fujita, Y. Ueda, S. Sato, H. Yokoyama, N. Mizuno, T. Kumasaka, M. Fujita, Self-assembly of $M_{30}L_{60}$ icosidodecahedron. *Chem* **1**, 91–101 (2016).

19. L. R. MacGillivray, J. L. Atwood, A chiral spherical molecular assembly held together by 60 hydrogen bonds. *Nature* **389**, 469–472 (1997).

20. Y. Z. Liu, C. H. Hu, A. Comotti, M. D. Ward, Supramolecular Archimedean cages assembled with 72 hydrogen bonds. *Science* **333**, 436–440 (2011).

21. L. Liang, W. Zhao, X. J. Yang, B. Wu, Anion-coordination-driven assembly. *Acc. Chem. Res.* **55**, 3218–3229 (2022).

22. L. Liang, P. R. Su, Y. Wang, B. Y. Li, S. Lu, H. W. Ma, Y. Y. Chen, W. Zhao, X. P. Li, X. J. Yang, B. Wu, Peripheral control of the assembly and chirality of anion-based octanuclear cubes by cation-π networks. *J. Am. Chem. Soc.* **146**, 10908–10916 (2024).

23. B. Y. Li, W. Y. Zhang, S. Lu, B. Zheng, D. Zhang, A. Y. Li, X. P. Li, X. J. Yang, B. Wu, Multiple transformations among anion-based $A_{2n}L_{3n}$ assemblies: Bicapped trigonal antiprism A_8L_{12}, tetrahedron A_4L_6, and triple Helicate A_2L_3 (A = Anion). *J. Am. Chem. Soc.* **142**, 21160–21168 (2020).

24. H. Wu, Y. Wang, L. Dordevic, P. Kundu, S. Bhunia, A. X. Y. Chen, L. Feng, D. K. Shen, W. Q. Liu, L. Zhang, B. Song, G. C. Wu, B. T. Liu, M. Y. Yang, Y. Yang, C. L. Stern, S. I. Stupp, W. A. Goddard, W. P. Hu, J. F. Stoddart, Dynamic supramolecular snub cubes. *Nature* **637**, 347–353 (2025).

25. M. Rossmann, R. Kuhn, W. Zhang, S. Pletnev, J. Corver, E. Lenches, C. Jones, S. Mukhopadhyay, P. Chipman, E. Strauss, T. Baker, J. Strauss, Structure of dengue virus: Implications for flavivirus organization, maturation, and fusion. *Cell* **108**, 717–725 (2002).

26. I. D. Brown, Anion-anion repulsion, coordination number, and the asymmetry of hydrogen bonds. *Can. J. Phys.* **73**, 676–682 (1995).

27. J. M. Lehn, Cryptates: The chemistry of macropolycyclic inclusion complexes. *Acc. Chem. Res.* **11**, 49–57 (1978).

28. K. Bowman-James, Alfred werner revisited: The coordination chemistry of anions. *Acc. Chem. Res.* **38**, 671–678 (2005).

29. Z. Zhuo, Z. A. Nan, W. Z. Fu, W. Wang, G. L. Li, M. Y. Wu, M. C. Hong, Y. G. Huang, Anion-coordination and π–π stacking interaction driven assembly of a complex Frank-Kasper structure. *Chem* **11**, 102371 (2025).

30. G. L. Li, Z. Zhuo, B. Wang, X. L. Cao, H. F. Su, W. Wang, Y. G. Huang, M. C. Hong, Constructing π-stacked supramolecular cage based hierarchical self-assemblies via π–π stacking and hydrogen bonding. *J. Am. Chem. Soc.* **143**, 10920–10929 (2021).

31. B. Wang, Z. A. Nan, J. Liu, Z. X. Lu, W. Wang, Z. Zhuo, G. L. Li, Y. G. Huang, Metalation of a hierarchical self-assembly consisting of π-stacked cubes through single-crystal-to-single-crystal transformation. *Molecules* **28**, 4923 (2023).

32. D. R. Walker, A. A. Alizadehmojarad, A. B. Kolomeisky, J. D. Hartgerink, Charge-free, stabilizing amide-π interactions can be used to control collagen triple-helix self-assembly. *Biomacromolecules* **22**, 2137–2147 (2021).

33. M. W. Krone, C. R. Travis, G. Y. Lee, H. J. Eckvahl, K. N. Kouk, M. L. Waters, More than π-π stacking: contribution of amide-π and CH-π interactions to crotonyllysine binding by the AF9 YEATS domain. *J. Am. Chem. Soc.* **142**, 17048–17056 (2020).

34. R. F. de Freitas, M. Schapira, A systematic analysis of atomic protein-ligand interactions in the PDB. *MedChemComm* **8**, 1970–1981 (2017).

35. S. Li, Q. Li, T. Chen, Z. Y. Ji, G. L. Li, M. Y. Wu, L. Y. Meng, Z. A. Nan, W. Wang, Z. Zhuo, F. R. Fan, Y. G. Huang, Temperature-dependent separation of CO_2 from light hydrocarbons in a porous self-assembly of vertexes sharing octahedra. *Adv. Sci.* **11**, 202308028 (2024).

36. X. N. Zhou, X. Yang, Z. X. Lu, Z. A. Nan, Y. G. Huang, A supramolecular octahedron assembled by anion coordination and π-π stacking interactions showing dual emission. *J. Mol. Struct.* **1305**, 137721 (2024).

37. Y. N. Imai, Y. Inoue, I. Nakanishi, K. Kitaura, Amide-π interactions between formamide and benzene. *J. Comput. Chem.* **30**, 2267–2276 (2009).

38. D. W. Heinz, W. A. Baase, F. W. Dahlquist, B. W. Matthews, How amino-acid insertions are allowed in an α-helix of T_4-lysozyme. *Nature* **361**, 561–564 (1993).

39. B. Hammouda, A new Guinier–Porod model. *J. Appl. Cryst.* **43**, 716–719 (2010).

40. P. Moore, Small-angle scatting information-content and error analysis. *J. Appl. Cryst.* **13**, 168–175 (1980).

41. J. Ilavsky, P. R. Jemian, Irena: Tool suite for modelling and analysis of small-angle scattering. *J. Appl. Cryst.* **42**, 347–353 (2009).

42. F. Zhang, J. Ilavsky, G. G. Long, J. P. Quintana, A. J. Allen, P. R. Jemian, Glassy carbon as an absolute intensity calibration standard for small-angle scattering. *Metall. Mater. Trans. A* **41**, 1151–1158 (2010).

43. A. Nelson, Co-refinement of multiple-contrast neutron/X-ray reflectivity data using MOTOFIT. *J. Appl. Cryst.* **39**, 273–276 (2006).

44. G. Beaucage, Approximations leading to a unified exponential/power-law approach to small-angle scattering. *J. Appl. Cryst.* **28**, 717–728 (1995).

45. C. J. Lu, M. M. Zhang, D. T. Tang, X. Z. Yan, Z. Y. Zhang, Z. X. Zhou, B. Song, H. Wang, X. P. Li, S. C. Yin, H. Sepehrpour, P. J. Stang, Fluorescent metallacage core supramolecular polymer gel formed by orthogonal metal coordination and host-guest interactions. *J. Am. Chem. Soc.* **140**, 7674–7680 (2023).

46. Z. E. Zhang, Y. F. Zhang, Y. Z. Zhang, H. L. Li, L. Y. Sun, L. J. Wang, Y. F. Han, Construction and hierarchical self-assembly of multifunctional coordination cages with triangular metal‒metal-bonded units. *J. Am. Chem. Soc.* **142**, 21160–21168 (2020).

47. S. R. Zhang, D. Y. Du, J. S. Qin, S. J. Bao, S. L. Li, W. W. He, Y. Q. Lan, P. Shen, Z. M. Su, A fluorescent sensor for highly selective detection of nitroaromatic explosives based on a 2D, extremely stable, metal-organic framework. *Chem. A Eur. J.* **20**, 3589–3594 (2014).

48. C. L. Chen, Q. Zhang, J. H. Yao, J. Y. Zhang, B. S. Kang, C. Y. Su, Assembly of 1D meso coordination polymer from a chiral mononuclear complex by N-deprotonation of the tris(2-benzimidazolyl) ligand. *Inorg. Chim. Acta* **361**, 2934–2940 (2008).

49. B. T. Chen, N. Morlanés, E. Adogla, K. Takanabe, V. O. Rodionov, An efficient and stable hydrophobic molecular cobalt catalyst for water electro-oxidation at neutral pH. *ACS Catal.* **6**, 4647–4652 (2016).

50. C. Nazikkol, R. Wegner, J. Bremer, B. Krebs, Tripodal ligands: Design of distorted coordination polyhedra in biomimetic metal complexes. Crystal structures of [Zn(SCN)(ntb)](SCN)·^{i}PrpOH and [Fe(acac)(ntb)]$(ClO_4)_2$·$2CH_2Cl_2$·^{i}PrpOH, ntb=N-tris(2-benzimidazolylmethyl)amine. *Z. Anorg. Allg. Chem.* **622**, 329–336 (1996).

51. G. M. Sheldrick, Crystal structure refinement with SHELXL. *Acta Cryst. C.* **71**, 3–8 (2015).

52. T. Li, D. Niu, L. Ji, Q. Li, B. Guan, H. Wang, G. Ouyang, M. Liu, Supramolecular rosette intermediated homochiral double helix. *Nat. Commun.* **16**, 1698 (2025).

53. R. Evans, Z. Deng, A. K. Rogerson, A. S. McLachlan, J. J. Richards, M. Nilsson, G. A. Morris, Quantitative interpretation of diffusion-ordered NMR spectra: Can we rationalize small molecule diffusion coefficients. *Angew. Chem. Int. Ed. Engl.* **52**, 3199–3202 (2013).

54. M. J. Frisch, G. W. Trucks, H. B. Schlegel, G. E. Scuseria, M. A. Robb, J. R. Cheeseman, G. Scalmani, V. Barone, G. A. Petersson, H. L. Nakatsuji, X. M. Caricato, A. V. Marenich, J. Bloino, B. G. Janesko, R. Gomperts, B. Mennucci, H. P. Hratchian, J. V. Ortiz, A. F. Izmaylov, J. L. Sonnenberg, Williams, F. Ding, F. Lipparini, F. Egidi, J. Goings, B. Peng, A. Petrone, T. Henderson, D. Ranasinghe, V. G. Zakrzewski, J. Gao, N. Rega, G. Zheng, W. Liang, M. Hada, M. Ehara, K. Toyota, R. Fukuda, J. Hasegawa, M. Ishida, T. Nakajima, Y. Honda, O. Kitao, H. Nakai, T. Vreven, K. Throssell, J. A. Montgomery Jr., J. E. Peralta, F. Ogliaro, M. J. Bearpark, J. J. Heyd, E. N. Brothers, K. N. Kudin, V. N. Staroverov, T. A. Keith, R. Kobayashi, J. Normand, K. Raghavachari, A. P. Rendell, J. C. Burant, S. S. Iyengar, J. Tomasi, M. Cossi, J. M. Millam, M. Klene, C. Adamo, R. Cammi, J. W. Ochterski, R. L. Martin, K. Morokuma, O. Farkas, J. B. Foresman, D. J. Fox, *Gaussian 16 Rev. B.01* (Gaussian Inc., 2016).

55. T. Lu, Q. X. Chen, Shermo: A general code for calculating molecular thermochemistry properties. *Comput. Theor. Chem.* **1200**, 113249 (2021).
